# Assessment of sediment physiochemical properties, microbial and predicted functional diversity in mangrove eco-restoration sites of Hamata, Mangrove Bay, and Saffaga along the Egyptian Red Sea coast

**DOI:** 10.1007/s11356-025-37234-1

**Published:** 2025-12-07

**Authors:** Muziri Mugwanya, Eric Zadok Mpingirika, Yasmine AbdelMaksoud, Rafat A. Eissa, Hani Sewilam

**Affiliations:** 1 Center for Applied Research On the Environment and Sustainability (CARES), School of Science and Engineering, The American University in Cairo, AUC Avenue, P.O. Box 74, New Cairo, 11835 Egypt; 2 Eppley Institute for Research in Cancer and Allied Diseases, University of Nebraska Medical Center, Omaha, NE 68198 USA; 3 UNESCO Chair in Hydrological Changes and Water Resources Management, RWTH Aachen University, Aachen, Germany

**Keywords:** Bacterial ecology, Blue carbon ecosystems, Carbon sequestration, Mangroves, Sustainability

## Abstract

Microbial communities perform important roles in nutrient cycling, degradation of environmental pollutants, and support of various life forms on Earth. Mangroves live in very harsh environments, and if not for the existence of several microbial species in their ecosystems, they would not survive. The Egyptian Red Sea coast is dominated by two mangrove species, *Avicennia marina* and *Rhizophora mucronata*, which serve as breeding grounds for marine organisms and aid in carbon sequestration. Despite their ecological significance, comparative studies examining the physiochemical properties and heavy metal concentration of mangrove sediments of two dominant species along the Egyptian Red Sea coast (Hamata, Mangrove Bay, and Saffaga) and their relationship to microbial and functional diversity are scarce. Our findings revealed significant differences in sodium ions, potassium ions, organic carbon, and bulk density at 30–50 cm depth across the locations. Heavy metal analysis revealed significantly lower concentrations of zinc and manganese and high concentrations of copper in sediment samples collected from Mangrove Bay at all sampling depths. Metagenomics analysis revealed that the dominant phyla across the three sites were *Pseudomonadota*, *Bacillota*, and *Bacteroidota*, along with *Actenomycetota*, and *Chloroflexota*, and unclassified bacteria. Within the phylum *Bacillota*, several major classes were identified, including *Bacillota_A_368345*, *Bacillota_I*, and *Bacillota_C*. Functional prediction revealed a higher abundance of microbes involved in energy metabolism and carbon cycle, whereas a lower abundance of microbes involved in sulfur and nitrogen cycles was noted across the sites. In conclusion, the identification of different microbial communities in sediments collected along the Egyptian Red Sea coastal areas suggests the role of different mangrove species and human activities in recruiting unique microbial species involved in promoting their survival under different environmental factors.

**Supplementary information:**

The online version contains supplementary material available at 10.1007/s11356-025-37234-1.

## Introduction

Mangroves are one of the world’s most productive ecosystems along the coastal part of the tropical and subtropical regions (Ghosh et al. 2022; Wainwright et al. 2023; Alghamdi et al. 2024). Their coverage is approximately 150,000 km^2^ across 123 countries, and they play a vital role in the habitation of various aquatic organisms, food supply, protection and stabilization of coastal areas, and aid in the phytoremediation of contaminated soils (Hossain et al. 2022; Baskaran et al. 2023; Farshid et al. 2023; Dajam et al. 2024; Karmakar et al. 2025). Their uptake of carbon dioxide (CO_2_) is estimated to be four times higher than that of inland terrestrial plants, and their annual carbon sequestration may reach up to 25.5 million tons (Patil et al. 2012; Ahmed et al. 2022; Chatting et al. 2022; Baskaran et al. 2023). The efficient carbon sequestration characteristic of mangroves is attributed to their high productivity and slow rates of decomposition of organic matter (OM) (Kida and Fujitake 2020; Guo et al. 2024), thus attracting the attention of the scientific community, which is keen on nature-based solutions for reducing greenhouse gases in the atmosphere.

Interestingly, mangrove ecosystems are rich in different microbial species (i.e., marine, freshwater, and terrestrial microbes) performing several biogeochemical processes such as carbon, sulfur, and nitrogen cycling and degradation of several organic and xenobiotic substances (Li et al. 2021a; Sarker et al. 2021; Padhy et al. 2022; Wang et al. 2022a; Ghose et al. 2024; Kannan et al. 2024). Moreover, the soil or sediment physiochemical properties, such as salinity, pH, electroconductivity (EC), organic carbon (OC), bulk density (BD), and elements or heavy metals (HMs) influence the activity of these microorganisms and the survival of mangrove forests (Lai et al. 2022; Li et al. 2022b). Hence, it is imperative to study the correlation between the soil or sediment’s physiochemical properties and microbial composition. The application of next-generation sequencing (NGS) technologies in studying microbial diversity and composition has generated useful information in the literature, where several cultured, uncultured, and novel microbial species have been identified and extensively studied (Alam et al. 2021; Park et al. 2021). This unbiased approach detects all the genomes present in a sample and has been widely used in environmental microbiology (Tan et al. 2015; Nafea et al. 2024). For instance, Hu et al. (2022) reported *Pseudomonadota*, *Chloroflexi*, *Bacillota*, and *Bathyarchaeota* as the dominant bacterial phyla found in soil samples of the mangroves in South China. Moreover, heavy metals such as chromium (Cr), zinc (Zn), copper (Cu), lead (Pb), and nickel (Ni) were the main factors influencing microbial diversity. From the Indian mangrove surface water, Ghosh et al. (2022) found that the *Pseudomonadota*, *Bacillota*, *Actinobacteria*, *Bacteroidota*, and *Cyanobacteria* were the dominant bacterial phyla present with Cu, Ni, and arsenic (As) resistance genes found in comparable abundances across the studied sites. Functional annotations revealed metabolic activities such as amino acids, carbohydrates, phosphorus, and nitrogen metabolisms to be uniformly distributed across the studied sites. A study on the *Avicennia marina* ecosystem along the Saudi Arabian Red Sea coastline revealed *Pseudomonadota*as the dominant phylum with functional prediction, indicating diverse microbial roles in HM uptake and plant growth promotion (Alghamdi et al. 2024). Moreover, a novel bacterial species, such as *Salinicola rhizosphaerae* (strain MSSRFH1^T^) has been identified from the rhizosphere of *A. marina* (Raju et al. 2016).

Based on NGS’s massive data generation, this study, therefore, employed Illumina NGS of the V3–V4 hypervariable region of the 16S rRNA to (i) elucidate the microbial composition and diversity of sediment samples collected around *Avicennia marina* and *Rhizophora mucronata* mangrove species at three coastal sites along the Egyptian Red Sea coast, (ii) correlate the microbial composition and diversity patterns with the detected heavy metals in sediment samples, and (iii) predict the function of the detected microbiota in heavy metal contaminated sites. This is the first comparative analysis linking sediment physiochemistry, heavy metal contamination, and microbial functional diversity in Egyptian Red Sea mangroves (*Avicennia marina* and *Rhizophora mucronata*), and the study results will provide new knowledge that will be helpful in mangrove ecosystem management, conservation, and restoration.

## Materials and methods

### Sample collection

**Fig. 1:**
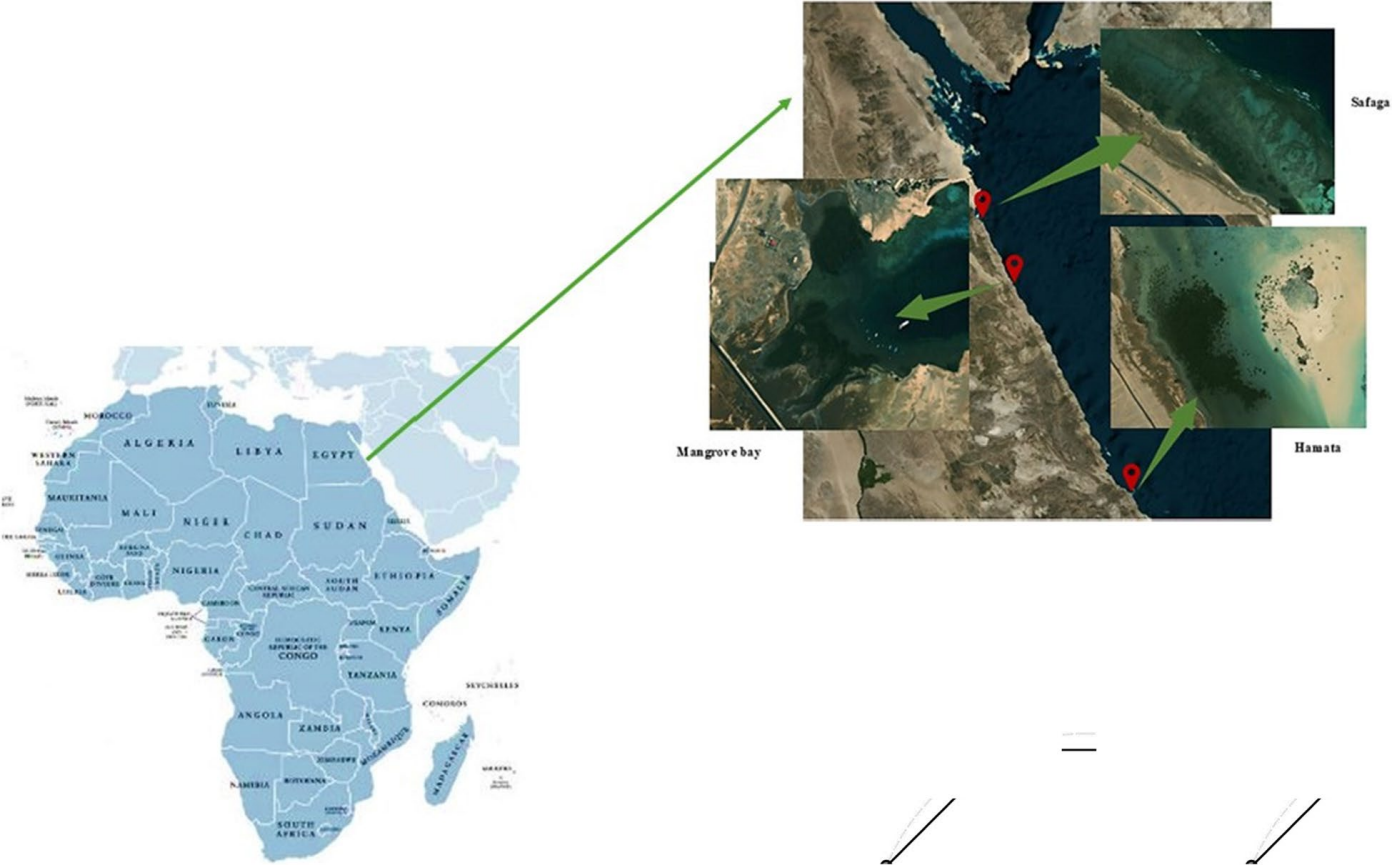
Map showing the geographical sites from which the sediment samples were collected along the Egyptian Red Sea coast

From 16th–19th March 2023, sediment samples were collected from three different locations along the Egyptian Red Sea coastline: Hamata (24°18′33.3″N, 35°21′43.64″E), Mangrove Bay (25°52′4.92″N, 34°24′55.99″E), and Saffaga (26°36′55.74″N, 34°0′46.17″E). Samples were coded as follows: HA, for sediment samples collected from Hamata around the *Avicennia marina* species; HR, for sediment samples collected from Hamata around the *Rhizophora mucronata* species; MA, for sediment samples collected from Mangrove Bay around the *A. marina* species; SA, for sediment samples collected from Saffaga around the *A. marina* species; and SR, for sediment samples collected from Saffaga around the *R. mucronata* species. The mangrove forests in these locations have been affected by different human activities, such as urbanization, agriculture, and tourism. Moreover, the mangrove site in Saffaga is near a mining port (Abu Tartour Port). As such, the Egyptian government has taken a keen interest in restoring mangroves in these sites. Figure 1 shows the geographical sites from which the sediment samples were collected. Sediment samples (3 replicates) were collected around two dominant mangrove species, *Avicennia marina* and *Rhizophora mucronata,* at different depths (0–15 cm, 15–30 cm, 30–50 cm, and 50–100 cm) using soil augers of different depths. A GPS land area measurement meter (model: Mingzhe, model number: BEIMQWE12670JK) was used to mark the sampling sites. A total of 60 samples (i.e., 24 from Hamata, 12 from Mangrove Bay, and 24 from Saffaga) were collected and put in labelled, sterilized plastic bags. These samples were immediately put on dry ice and kept frozen until further analysis.

### Sediment sample analysis

At each sampling location (Hamata, Mangrove Bay, and Saffaga), sediment samples were collected and sectioned into four depth intervals: (0–15 cm, 15–30 cm, 30–50 cm, and 50–100 cm). For each depth interval, the collected sediment was thoroughly homogenized to create a composite sample representative of the specific depth at that location. From this composite sample, three subsamples (replicates) were taken for subsequent physiochemical analysis at the Soil and Water Research Institute of the Agricultural Research Center (ARC) in Giza, Egypt, and heavy metal analysis. Briefly, 2 g of the sediment sample per replicate was air-dried and sieved through a 10-mesh sieve with a 2 mm aperture size (USA Standard Sieve). The soil pH and electroconductivity (EC) were measured as previously described by Sewilam et al. (2023). Organic carbon was determined as described by Dookie et al. (2024) with slight modifications. Briefly, 2 g of the sediment sample was used for organic carbon estimation. Twenty milliliters of potassium dichromate was added to the sediment sample, and the mixture was stirred for 1 min, followed by the addition of 40 ml of concentrated sulfuric acid. The mixture was left to stand for 30 min, and 400 ml of distilled water was added. Three drops of phenolphthalein indicator were added, and the mixture was titrated with 0.5 M ferrous sulfate until color changes. Organic carbon was calculated based on the equation below.

$$\mathrm{Organic}\;\mathrm{carbon}=\left(\mathrm{ml}\;\mathrm{of}\;\mathrm{blank}-\mathrm{ml}\;\mathrm{of}\;\mathrm{determination}\right)\;\times\;0.399$$

Sediment cations and anions were measured according to the methods described by Madeira et al. (2003). For the sediment bulk density, this was determined as previously described by Doran and Mielke (1984). Furthermore, the concentration of different heavy metals was analyzed using inductively coupled plasma (ICP) spectrometry (model Ultima 2 JY Plasma) (Soltanpour 1991).

### DNA extraction and sequencing

Sediment samples were transported on dry ice to the laboratory and immediately stored under −20 °C until further analysis. Sediment samples from a 0 to 15 cm depth per replicate were homogenized under sterilized conditions. Then, 400 mg of the sediment sample was used for total genomic DNA extraction using the innuSPEED Soil DNA Kit 2.0 (LOT No. 004–23), following the manufacturer’s instructions. DNA concentration was determined by using the NanoDrop™ One/One^C^ Microvolume UV–Vis Spectrophotometer from Thermo Fisher Scientific. Purity and completeness were evaluated using 1% agarose gel electrophoresis.

The 16S rRNA genes were amplified using the specific primers for V3 and V4 hypervariable regions: 341 F (CCTAYGGGRBGCASCAG) and 806R (GGACTCANNGGGTATCTAAT) (Wu et al. 2024). PCR was performed in a 30 µL reaction with 15 µl Master Mix (New England Biolabs, USA), 0.2 µM each of forward and reverse primers and 10 ng of template DNA. The thermocycling conditions were as follows: pre-degeneration at 98 ℃ for 1 min, 30 cycles of denaturation at 98 °C for 10 s, annealing at 50 °C for 30 s, and extension at 72 ℃ for 30 s, followed by a final extension at 72 ℃ for 5 min. Electrophoretic detection was performed on a 2% agarose gel. Samples with a bright main band between 400 and 450 bp were selected for subsequent experiments. PCR products were purified using a kit (Tiangen Biotech, China). The purified products were used to prepare the library. Sequencing libraries were generated using the TIANSeq Fast DNA Library Prep Kit (Tiangen Biotech, China). Library quality was assessed on a Qubit 2.0 fluorometer (Thermo Scientific) and an Agilent 2100 Bioanalyzer. Finally, the libraries were sequenced on the Illumina platform using a 2 × 250 bp paired-end protocol.

### 16S rRNA data processing and analysis

We used the Qiime2 pipeline (v2024.10) to process raw data (Bolyen et al. 2019). VSEARCH was used for denoising, chimera filtering and clustering. The quality of the processed sequences is presented in Supplementary Fig. 1. Filtered sequences were clustered by 97% similarity into operational taxonomic units (OTUs) with cluster-features-open-reference parameters against Greengenes2 (v2024.9) (Rognes et al. 2016; McDonald et al. 2024). Taxonomic assignment for each clustered OTU was performed using the Naive Bayes classifier trained against the Greengenes2 database. We used the microeco R package (v1.5.0) to calculate alpha and beta diversity and visualization. Functional prediction for the abundance of communities was carried out using FAPROTAX (Louca et al. 2016) and Tax4Fun2 (Wemheuer et al. 2020) databases. Raw sequence reads have been deposited in the Sequence Read Archive (SRA) of the National Center for Biotechnology Information (NCBI) under a BioProject accession identification number PRJNA1224588.

### Statistical analysis

All datasets on the sediment physiochemical properties were subjected to normality and equality of variances tests using QQ plots and Levene’s test, respectively. One-way analysis of variance (ANOVA) was conducted to determine significant differences in the measured parameters at *p* < 0.05. The Duncan Multiple Range test (DMRT) was used as a post hoc test to detect differences in the sediment sample means. Visualization of results was performed in R statistical programming language (version 4.3.2).

## Results

### Physiochemical properties of sediment samples

Table 1 presents the results of the physiochemical properties of sediment samples collected at different depths. No significant differences in the pH, electroconductivity (EC), sulfate ions (SO4^2−^), bicarbonate ions (HCO_3_^−^), chloride ions (Cl^−^), sodium ions (Na^+^), potassium ions (K^+^), magnesium ions (Mg^2+^), calcium ions (Ca^2+^), organic carbon (OC), and bulk density (BD) were noted across the sediment samples collected at 0–15 cm, 15–30 cm, and 50–100 cm depths. However, MA significantly (*p* < 0.05) recorded higher pH values (8.31) than other sediment samples. Likewise, HR significantly recorded (*p* < 0.05) lower values for the EC (10.42 dS/m), Na^+^ (46.53 meq/L), and Ca^2+^ (30.50 meq/L) compared to SA and SR at 30–50 cm depth. The OC content was significantly (*p* < 0.05) higher in HA (0.64%) and SR (0.74%) than in other sediment samples at 30–50 cm depth.

**Table 1:** Physicochemical properties of sediment samples collected at different depths

			Anions (meq/L)			Cations (meq/L)					
Sample	pH (1:2.5)	EC (dS/m)	SO4^2−^	HCO_3_^−^	Cl^−^	Na^+^	K^+^	Mg^2+^	Ca^2+^	OC (%)	BD (g/cm^3^)
0–15 cm											
HA	8.17^a^ ± 0.04	15.01^a^ ± 1.50	67.15^a^ ± 17.19	6.17^a^ ± 1.15	94.5^a^ ± 27.07	74.93^a^ ± 20.98	1.88^a^ ± 0.12	36.83^a^ ± 10.69	54.17^a^ ± 14.57	0.49^a^ ± 0.04	1.31^a^ ± 0.05
HR	8.26^a^ ± 0.04	13.87^a^ ± 1.91	55.28^a^ ± 8.52	5.83^a^ ± 0.58	77.50^a^ ± 10.54	58.05^a^ ± 4.68	1.90^a^ ± 0.09	30.83^a^ ± 5.69	47.50^a^ ± 8.54	0.63^a^ ± 0.24	1.31^a^ ± 0.02
MA	8.33^a^ ± 0.11	19.37^a^ ± 3.48	82.62^a^ ± 11.87	7.17^a^ ± 0.58	101.83^a^ ± 21.94	74.02^a^ ± 6.61	2.63^a^ ± 0.58	49.83^a^ ± 17.21	65.83^a^ ± 11.50	0.39^a^ ± 0.20	1.46^a^ ± 0.09
SA	8.23^a^ ± 0.04	18.47^a^ ± 4.36	75.01^a^ ± 14.08	6.50^a^ ± 1.00	100.50^a^ ± 27.84	77.55^a^ ± 18.28	1.80^a^ ± 0.61	42.83^a^ ± 10.50	62.50^a^ ± 14.73	0.23^a^ ± 0.16	1.34^a^ ± 0.08
SR	8.03^a^ ± 0.26	19.05^a^ ± 3.13	70.45^a^ ± 9.01	6.83^a^ ± 1.15	113.17^a^ ± 21.36	81.65^a^ ± 17.86	1.50^a^ ± 0.43	45.83^a^ ± 7.37	61.50^a^ ± 6.93	0.56^a^ ± 0.06	1.34^a^ ± 0.08
15–30 cm											
HA	8.17^a^ ± 0.04	15.01^a^ ± 1.50	67.15^a^ ± 17.19	6.17^a^ ± 1.15	94.5^a^ ± 27.07	74.93^a^ ± 20.98	1.88^a^ ± 0.12	36.83^a^ ± 10.69	54.17^a^ ± 14.57	0.49^a^ ± 0.04	1.31^a^ ± 0.05
HR	8.26^a^ ± 0.04	13.87^a^ ± 1.91	55.28^a^ ± 8.52	5.83^a^ ± 0.58	77.50^a^ ± 10.54	58.05^a^ ± 4.68	1.90^a^ ± 0.09	30.83^a^ ± 5.69	47.50^a^ ± 8.54	0.63^a^ ± 0.24	1.31^a^ ± 0.02
MA	8.33^a^ ± 0.11	19.37^a^ ± 3.48	82.62^a^ ± 11.87	7.17^a^ ± 0.58	101.83^a^ ± 21.94	74.02^a^ ± 6.61	2.63^a^ ± 0.58	49.83^a^ ± 17.21	65.83^a^ ± 11.50	0.39^a^ ± 0.20	1.46^a^ ± 0.09
SA	8.23^a^ ± 0.04	18.47^a^ ± 4.36	75.01^a^ ± 14.08	6.50^a^ ± 1.00	100.50^a^ ± 27.84	77.55^a^ ± 18.28	1.80^a^ ± 0.61	42.83^a^ ± 10.50	62.50^a^ ± 14.73	0.23^a^ ± 0.16	1.34^a^ ± 0.08
SR	8.03^a^ ± 0.26	19.05^a^ ± 3.13	70.45^a^ ± 9.01	6.83^a^ ± 1.15	113.17^a^ ± 21.36	81.65^a^ ± 17.86	1.50^a^ ± 0.43	45.83^a^ ± 7.37	61.50^a^ ± 6.93	0.56^a^ ± 0.06	1.34^a^ ± 0.08
30–50 cm											
HA	8.16^b^ ± 0.06	14.74^ab^ ± 3.27	71.41^a^ ± 13.97	7.17^a^ ± 1.15	86.50^a^ ± 15.87	68.38^bc^ ± 9.63	2.73^ab^ ± 0.48	39.50^a^ ± 7.21	54.50^ab^ ± 13.08	0.64^a^ ± 0.16	1.30^b^ ± 0.04
HR	8.16^b^ ± 0.05	10.42^b^ ± 2.04	41.52^a^ ± 12.52	5.50^a^ ± 1.73	65.83^a^ ± 20.98	46.53^c^ ± 12.89	1.98^bc^ ± 0.54	27.17^a^ ± 10.26	30.50^b^ ± 1.00	0.37^b^ ± 0.08	1.29^b^ ± 0.23
MA	8.31^a^ ± 0.08	18.62^ab^ ± 3.73	74.78^a^ ± 16.99	7.50^a^ ± 0.00	103.88^a^ ± 23.09	76.75^ab^ ± 16.15	3.02^a^ ± 0.29	43.50^a^ ± 7.81	52.83^ab^ ± 8.50	0.31^b^ ± 0.00	1.48^a^ ± 0.11
SA	8.17^b^ ± 0.06	22.53^a^ ± 1.15	83.95^a^ ± 2.65	7.50^a^ ± 0.00	131.83^a^ ± 9.07	95.97^a^ ± 5.09	1.32^c^ ± 0.06	53.50^a^ ± 1.00	74.50^a^ ± 5.57	0.20^b^ ± 0.06	1.40^ab^ ± 0.02
SR	8.14^b^ ± 0.06	20.44^a^ ± 8.13	77.53^a^ ± 28.72	7.50^a^ ± 1.32	127.67^a^ ± 56.87	85.63^ab^ ± 19.97	2.25^ab^ ± 0.52	48.83^a^ ± 26.54	68.83^a^ ± 24.95	0.74^a^ ± 0.19	1.30^b^ ± 0.08
50–100 cm											
HA	8.20^a^ ± 0.11	20.83^a^ ± 1.93	71.70^a^ ± 22.31	6.50 ± 1.00	96.50^a^ ± 30.51	66.95^a^ ± 12.65	1.73^a^ ± 0.55	39.50^a^ ± 13.75	57.50^a^ ± 16.52	0.32^a^ ± 0.22	1.28^a^ ± 0.00
HR	8.22^a^ ± 0.04	13.85^a^ ± 5.79	51.82^a^ ± 19.45	5.83 ± 1.53	80.83^a^ ± 37.21	61.23^a^ ± 24.72	1.92^a^ ± 0.29	31.50^a^ ± 15.87	43.50^a^ ± 18.33	0.29^a^ ± 0.14	1.32^a^ ± 0.06
MA	8.30^a^ ± 0.10	18.84^a^ ± 2.69	82.35^a^ ± 14.38	7.50 ± 1.00	98.50^a^ ± 12.53	77.70^a^ ± 10.61	2.65^a^ ± 0.65	46.50^a^ ± 7.21	61.50^a^ ± 9.64	0.43^a^ ± 0.00	1.45^a^ ± 0.13
SA	8.20^a^ ± 0.07	21.16^a^ ± 3.98	87.88^a^ ± 23.21	7.50 ± 1.00	116.67^a^ ± 21.83	86.77^a^ ± 15.41	1.78^a^ ± 0.32	54.17^a^ ± 12.66	68.83^a^ ± 11.93	0.27^a^ ± 0.13	1.41^a^ ± 0.05
SR	8.21^a^ ± 0.04	17.11^a^ ± 2.67	69.72^a^ ± 10.86	9.50 ± 1.00	94.50^a^ ± 19.32	74.28^a^ ± 14.84	2.24^a^ ± 1.11	38.17^a^ ± 5.13	56.17^a^ ± 7.77	0.47^a^ ± 0.15	1.39^a^ ± 0.01

Tabular data is presented as mean ± SD. Different lower superscript letters in each column indicate significant differences at *p* < 0.05. *EC* electro conductivity, *SO4*^*2+*^ sulfate ions, *HCO*_*3*_^*−*^ hydrogen carbonate ions, *Cl*^*−*^ chloride ions, *Na*^*+*^ sodium ions, *K*^*+*^ potassium ions, *Mg*^*2+*^ magnesium ions, *Ca*^*2+*^ calcium ions, *OC* soil organic carbon, *BD* bulk density. Location and mangrove species: HA (Hamata, *Avicennia marina*), HR (Hamata, *Rhizophora mucronata*), MA (Mangrove Bay, *Avicennia marina*), SA (Saffaga, *Avicennia marina*), SR (Saffaga, *Rhizophora mucronata*)

### Heavy metal concentrations in sediment samples

The concentration of different heavy metals was assessed, and the results are presented in Table 2. MA sediment samples showed significantly (*p* < 0.05) higher concentrations of Cu across all sampling depths compared to all other sites. Conversely, the MA samples had significantly (*p* < 0.05) the lowest Cd concentrations at the 30–50 cm depth. At the 0–15 cm depth, MA (0.35 ppm) was significantly higher than all other sites (0.04–0.05 ppm). For cobalt (Co), significantly lower concentrations were noted in MA sediment samples at 0–15 cm, 30–50 cm, and 50–100 cm. No significant differences in the concentration of lead (Pb) and iron (Fe) were observed in all sediment samples across all depths except at 0–15 cm for Fe. For manganese (Mn), MA significantly (*p* < 0.05) recorded the lowest concentrations at 15–30 cm and 50–100 cm depths compared to other sediment samples. Similarly, MA significantly (*p* < 0.05) recorded the lowest values for zinc (Zn) at 15–30 cm and 30–50 cm depths compared to other sediment samples.

**Table 2:** The concentration of heavy metals in different sediment samples at different depths

Sample	Cu (ppm)	Cd (ppm)	Co (ppm)	Pb (ppm)	Fe (ppm)	Mn (ppm)	Zn (ppm)
0–15 cm							
HA	0.05^b^ ± 0.01	0.05^b^ ± 0.01	1.87^b^ ± 0.35	1.20^a^ ± 0.10	9.27^a^ ± 0.58	1.44^a^ ± 0.04	0.73^a^ ± 0.27
HR	0.04^b^ ± 0.01	0.04^b^ ± 0.01	1.13^c^ ± 0.06	0.57^a^ ± 0.32	9.26^a^ ± 1.41	1.48^a^ ± 0.07	0.59^ab^ ± 0.14
MA	0.35^a^ ± 0.05	0.35^a^ ± 0.05	2.25^b^ ± 0.08	0.53^a^ ± 0.14	7.03^b^ ± 0.70	1.22^a^ ± 0.34	0.34^b^ ± 0.01
SA	0.05^b^ ± 0.01	0.05^b^ ± 0.01	3.20^a^ ± 0.62	4.33^a^ ± 2.81	10.00^a^ ± 0.94	1.43^a^ ± 0.08	0.81^a^ ± 0.04
SR	0.05^b^ ± 0.01	0.05^b^ ± 0.01	3.67^a^ ± 0.21	3.27^a^ ± 2.25	9.43^a^ ± 0.74	1.40^a^ ± 0.35	0.79^a^ ± 0.12
15–30 cm							
HA	0.04^b^ ± 0.01	0.04^b^ ± 0.01	2.03^b^ ± 1.50	3.30^a^ ± 2.91	8.82^a^ ± 0.54	1.50^a^ ± 0.02	0.61^b^ ± 0.15
HR	0.04^b^ ± 0.01	0.04^b^ ± 0.01	1.53^b^ ± 0.65	3.37^a^ ± 3.42	9.87^a^ ± 0.18	1.54^a^ ± 0.12	0.62^ab^ ± 0.03
MA	0.33^a^ ± 0.10	0.33^a^ ± 0.10	2.26^b^ ± 0.08	0.44^a^ ± 0.28	7.58^a^ ± 1.51	1.04^b^ ± 0.36	0.40^c^ ± 0.03
SA	0.04^b^ ± 0.01	0.04^b^ ± 0.01	2.37^b^ ± 0.35	2.37^a^ ± 1.85	10.25^a^ ± 1.19	1.49^a^ ± 0.13	0.76^a^ ± 0.02
SR	0.05^b^ ± 0.01	0.05^b^ ± 0.01	3.93^a^ ± 0.42	1.27^a^ ± 0.64	9.63^a^ ± 1.36	1.54^a^ ± 0.07	0.74^ab^ ± 0.08
30–50 cm							
HA	0.05^b^ ± 0.01	0.63^a^ ± 0.25	2.17^b^ ± 0.81	1.30^a^ ± 0.69	10.08^a^ ± 0.92	1.39^a^ ± 0.24	0.69^ab^ ± 0.07
HR	0.06^b^ ± 0.01	0.80^a^ ± 0.61	1.17^c^ ± 0.31	2.70^a^ ± 2.86	9.60^a^ ± 0.59	1.39^a^ ± 0.18	0.59^b^ ± 0.05
MA	0.35^a^ ± 0.03	0.01^b^ ± 0.00	2.32^b^ ± 0.09	0.51^a^ ± 0.25	7.67^a^ ± 2.31	0.99^a^ ± 0.19	0.34^c^ ± 0.05
SA	0.06^b^ ± 0.01	0.50^ab^ ± 0.20	2.63^b^ ± 0.25	3.33^a^ ± 1.99	10.62^a^ ± 0.69	1.39^a^ ± 0.04	0.61^b^ ± 0.09
SR	0.04^b^ ± 0.01	0.90^a^ ± 0.20	4.13^a^ ± 0.40	0.70^a^ ± 0.53	9.45^a^ ± 0.61	1.39^a^ ± 0.11	0.75^a^ ± 0.04
50–100 cm							
HA	0.05^b^ ± 0.02	1.03^a^ ± 0.75	2.17^b^ ± 0.81	2.97^a^ ± 3.25	9.73^a^ ± 0.76	1.51^a^ ± 0.14	0.58^a^ ± 0.08
HR	0.05^b^ ± 0.01	0.40^a^ ± 0.10	1.17^c^ ± 0.31	0.84^a^ ± 0.75	9.75^a^ ± 0.95	1.37^a^ ± 0.11	0.53^a^ ± 0.18
MA	0.37^a^ ± 0.14	0.01^a^ ± 0.01	2.32^b^ ± 0.09	0.68^a^ ± 0.26	10.49^a^ ± 0.83	0.92^b^ ± 0.26	0.36^a^ ± 0.04
SA	0.04^b^ ± 0.00	0.60^a^ ± 0.10	2.63^b^ ± 0.25	3.00^a^ ± 2.25	10.78^a^ ± 0.83	1.49^a^ ± 0.19	0.63^a^ ± 0.12
SR	0.05^b^ ± 0.01	0.83^a^ ± 0.50	4.13^a^ ± 0.40	0.57^a^ ± 0.29	10.52^a^ ± 1.29	1.53^a^ ± 0.21	0.65^a^ ± 0.08

Tabular data is presented as mean ± SD. Different lower superscript letters in each column indicate significant differences at *p* < 0.05. Heavy metals: *Cu* copper, *Cd* cadmium, *Co* cobalt, *Pb* lead, *Fe* iron, *Mn* manganese, *Zn* zinc

### Principal component analysis

**Fig. 2:**
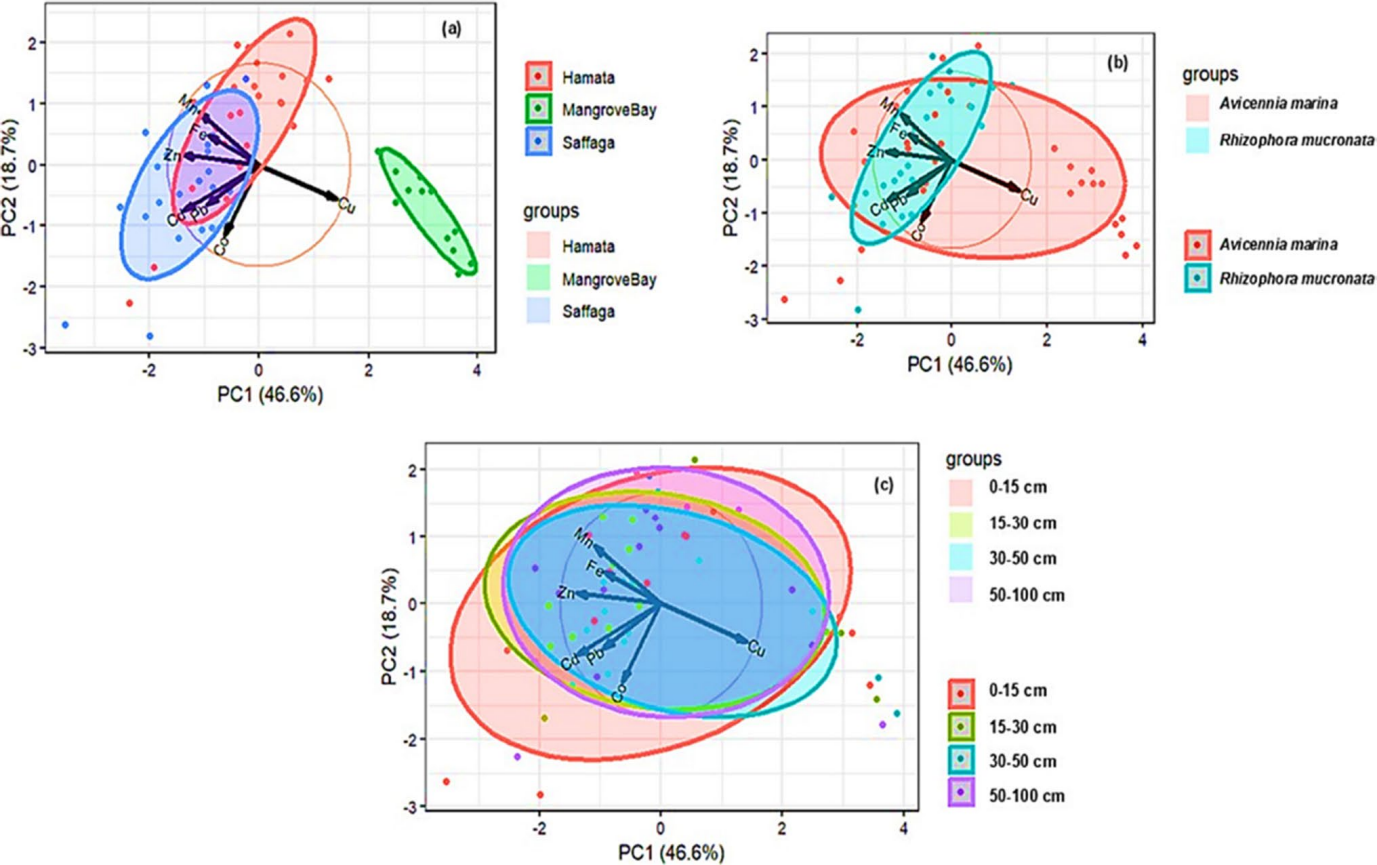
Principal component analysis (PCA) showing similarities and differences in heavy metal concentrations in sediment samples collected from **a** different locations, **b** mangrove species, and **c** depths

The principal component analysis (PCA) was computed to elucidate the associations among the HMs to reveal that a considerable portion of the observed variability (65.3%) could be explained by the first two components (Fig. 2). Based on the PCA results, the contribution of the first and second axes to the HM’s variance was found to be 46.6% and 18.7%, respectively. Moreover, the results indicate that iron (Fe) and manganese (Mn) are positively correlated and not correlated with zinc (Zn). Furthermore, copper (Cu) is negatively correlated with all the other HMs.

As indicated by the PCA biplots, there was an overlap of the biplots of Saffaga and Hamata, thus indicating similarities in the concentration of HMs in the sediments, unlike Mangrove Bay (Fig. 2a). Furthermore, there was an overlap of biplots for the two mangrove species, *A. marina* and *R. mucronata* (Fig. 2b), as well as an overlap of biplots for the different depths (Fig. 2c), which indicates similarities in HM concentrations in sediment samples obtained from such sources.

### Relative abundance, unique, and shared OTUs

**Fig. 3:**
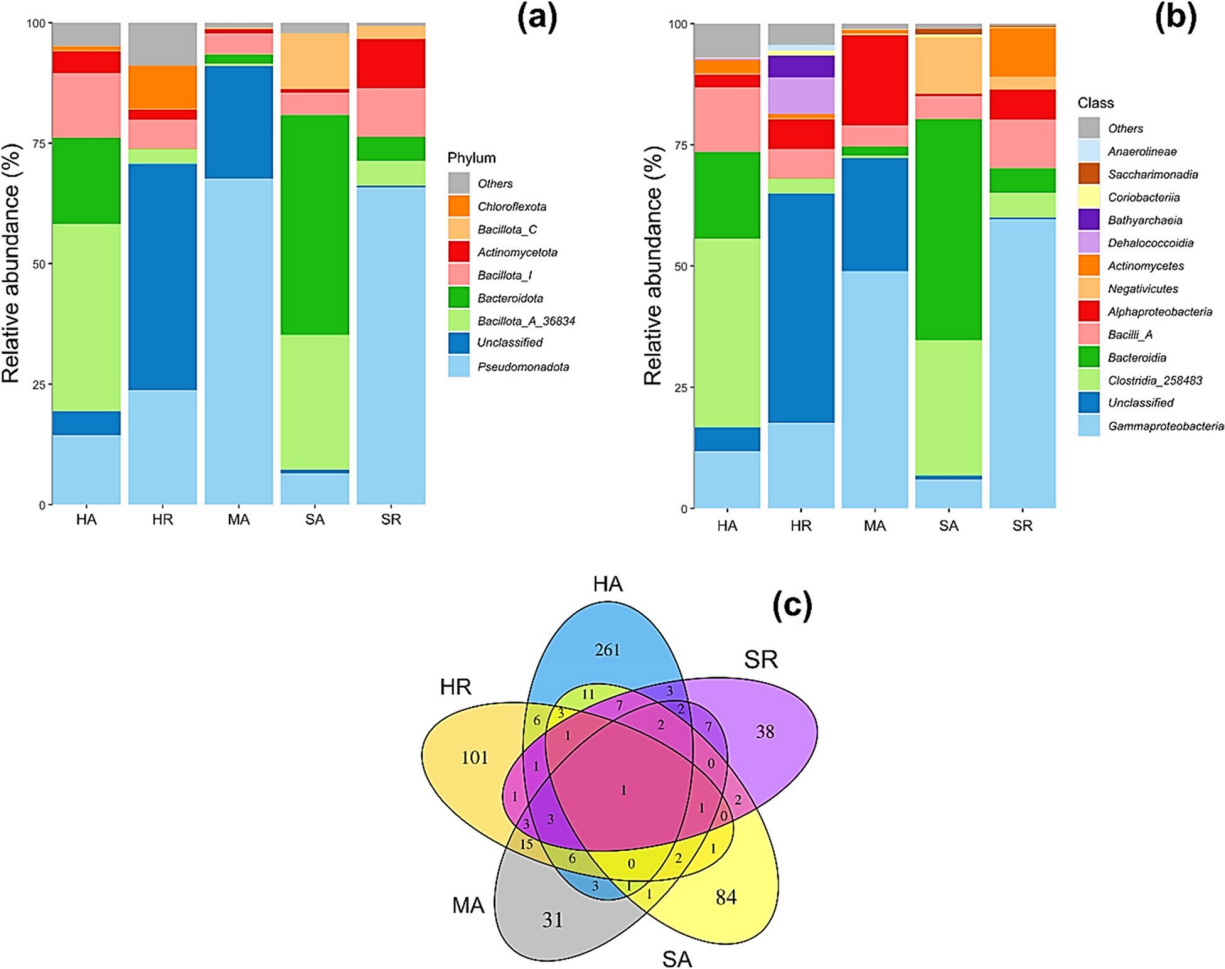
Relative abundance of dominant **a** phyla and **b** classes of bacteria identified through the 16S rRNA gene V3-V4 hypervariable region annotated through the Greengenes2 database; **c** Venn diagram of unique and shared operational taxonomic units (OTUs). Locations and mangrove species: HA (Hamata, *Avicennia marina*), HR (Hamata, *Rhizophora mucronata*), MA (Mangrove Bay, *Avicennia marina*), SA (Saffaga, *Avicennia marina*), SR (Saffaga, *Rhizophora mucronata*)

The relative abundance of dominant bacteria at the phylum and class levels is shown in Fig. 3a and b, respectively. At the phylum level, *Pseudomonadota*,* Bacillota*, and *Bacteroidota* were dominant across the sites, though their proportions varied. In HA, *Bacillota* was most abundant, followed by *Bacteroidota* (17.9%) and *Pseudomonadota* (14.4%). In contrast, HR was dominated by unclassified bacteria (49.2%) and *Pseudomonadota* (24.9%). MA was heavily dominated by *Pseudomonadota* (67.6%), while SA showed a high abundance of *Bacteroidota* (45.9%) and *Bacillota* (44.4%). SR was also dominated by *Pseudomonadota* (65.9%).

At the class level (Fig. 3b), *Clostridia* (37%), *Bacteroidia* (18%), *Bacilli* (13.3%), and *Gammaproteobacteria* (11.7%) were the dominant classes in HA sediment samples. In contrast, the unclassified bacteria (49.2%), *Gammaproteobacteria* (18.5%), *Dehalococcoidia* (7.9%), *Alphaproteobacteria* (6.4%), and *Bacilli_A* (6.3%) were the dominant classes in HR sediment samples. For MA, the dominant classes were *Gammaproteobacteria* (48.9%), unclassified bacteria (23.3%), and *Alphaproteobacteria* (18.7%). SA sediment samples contained *Bacteroidia* (45.9%), *Clostridia_258483* (28.1%), *Negativicutes* (11.6%), *Gammaproteobacteria* (6%), and *Bacilli* (4.7%) as the dominant classes. In contrast, *Gammaproteobacteria* (59.7%), *Bacilli* (10%), *Actinomycetes* (10%), *Clostridia* (5.2%), and *Alphaproteobacteria* (6.2%) were the dominant classes in SR sediment samples.

Figure 3c shows the unique and shared operational taxonomic units (OTUs) identified from sediment samples collected around *A. marina* and *R. mucronata* at different locations. Unique OTUs in HA were 261, 101 in HR, 31 in MA, 84 in SA, and 38 in SR. Only one OTU was shared among all the site locations and mangrove species. 11 OTUs were shared between HA and HR, 3 between HA and SR, 6 between HA and MA, 7 between HA and SA, 7 between HR and SR, 15 between HR and MA, 6 between HR and SA, 2 between SR and MA, 2 between SR and SA, 2 between SA and MA, 7 among HA, HR, and SR, 3 among HA, HR, and MA, and 3 among HR, SR, and MA.

### Alpha and beta diversity

**Fig. 4:**
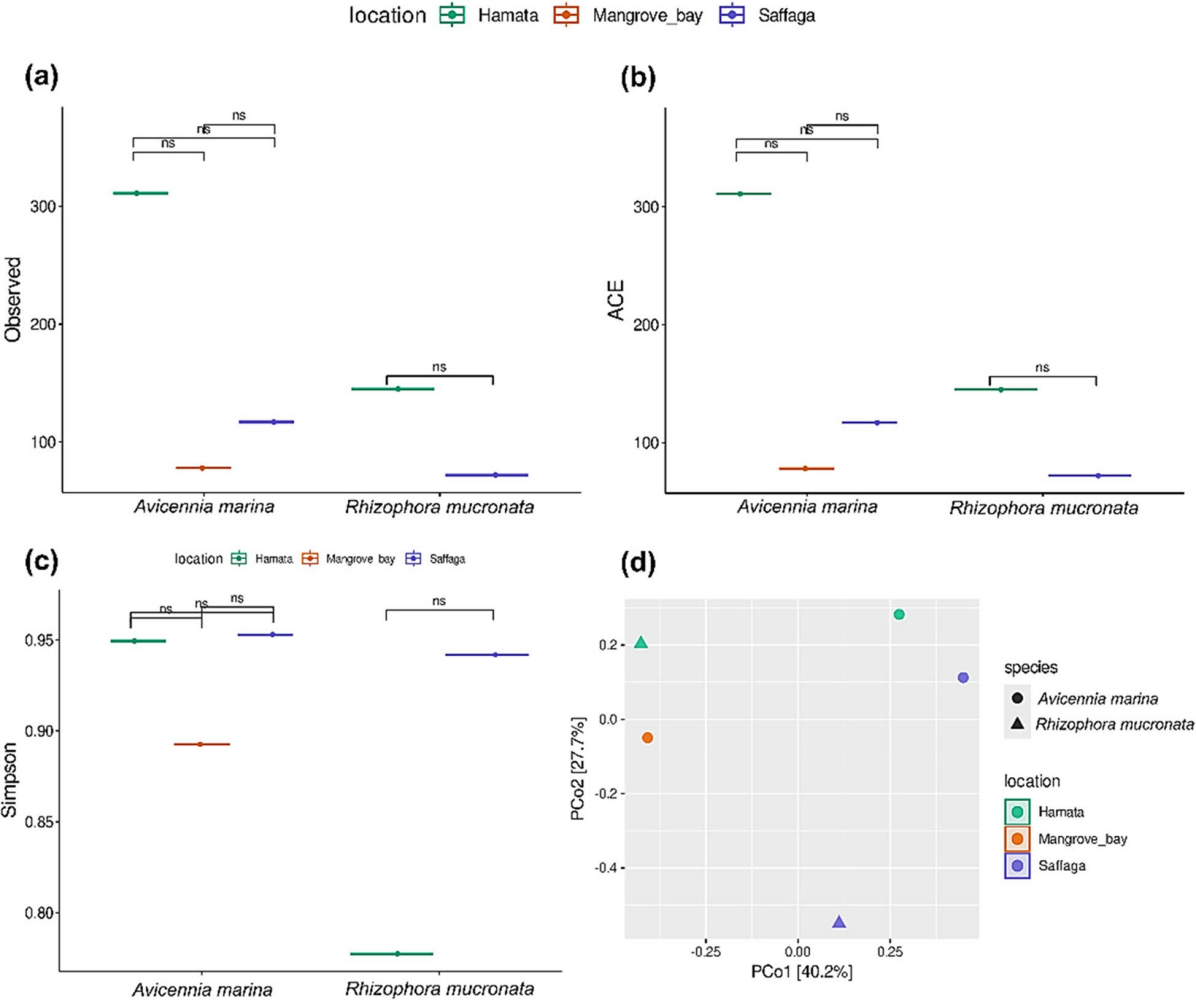
Alpha and beta diversity plots. **a** Plots corresponding to the Observed index, **b** plots corresponding to the ACE index, **c** plots corresponding to the Simpson index, and **d** principal coordinate analysis corresponding to the Bray–Curtis dissimilarity index (beta diversity). Statistical analysis using the Kruskal–Wallis test with Benjamini–Hochberg correction for multiple comparisons (false discovery rate); ns, not significant

The alpha and beta diversity indices are presented in Fig. 4a and b, respectively. Based on the data obtained concerning alpha diversity (Observed, ACE, and Simpson), no significant differences were noted between locations and mangrove species. Beta diversity indicated no clustering of the locations and mangrove species, thus indicating differences in the microbial composition between the locations and mangrove species. Moreover, PERMANOVA analysis indicated no significant differences in the locations’ dissimilarity.

### Redundancy discrimination analysis

**Fig. 5:**
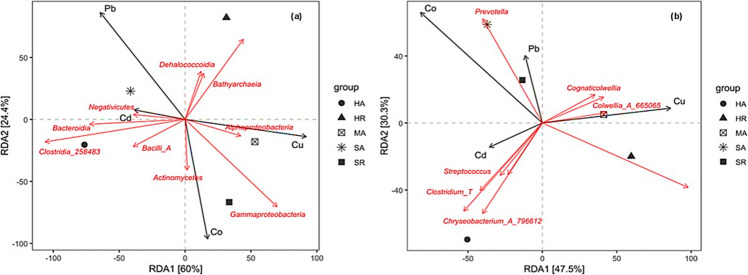
Redundancy discrimination analysis depicting the relationship between environmental variables and **a** classes and **b** genera of bacteria in different locations and mangrove species. Environmental variables that significantly explained the variability in the composition of the microbial community were fitted to the ordination. Arrows indicate the magnitude and direction of environmental variables associated with different bacterial classes and genera. Locations and mangrove species: HA (Hamata, *Avicennia marina*), HR (Hamata, *Rhizophora mucronata*), MA (Mangrove Bay, *Avicennia marina*), SA (Saffaga, *Avicennia marina*), SR (Saffaga, *Rhizophora mucronata*). Heavy metals: Cu, copper; Pb, lead; Fe, iron; Mn, manganese; Zn, zinc; Cd, cadmium; and Co, cobalt

With a selection for the significant variables that influence microbial composition, we used the redundancy discrimination analysis (RDA) to depict the relationship between environmental variables and the composition of the microbial community at different locations and mangrove species is presented in Fig. 5. The RDA showed that microbial communities were different as per the relationship with the environmental variables. At the class level (Fig. 5a), *Negativicutes* were positively correlated with Cd in sediment samples collected around *A. marina* in Saffaga, whereas *Alphaproteobacteria* and *Actinomycetes* were positively correlated with Cu and Co in sediment samples collected around *A. marina* and *R. mucronata* in Mangrove Bay and Saffaga, respectively. At the genus level (Fig. 5b), *Streptococcus*, *Clostridium_T*, and *Chryseobacterium_A_796612* were positively correlated with Cd in sediment samples collected around *A. marina* in Hamata. Furthermore, *Cognaticolwellia* and *Colwellia_A_665065* were positively correlated with Cu in sediment samples collected around *A. marina* in Mangrove Bay.

### Functional prediction

**Fig. 6:**
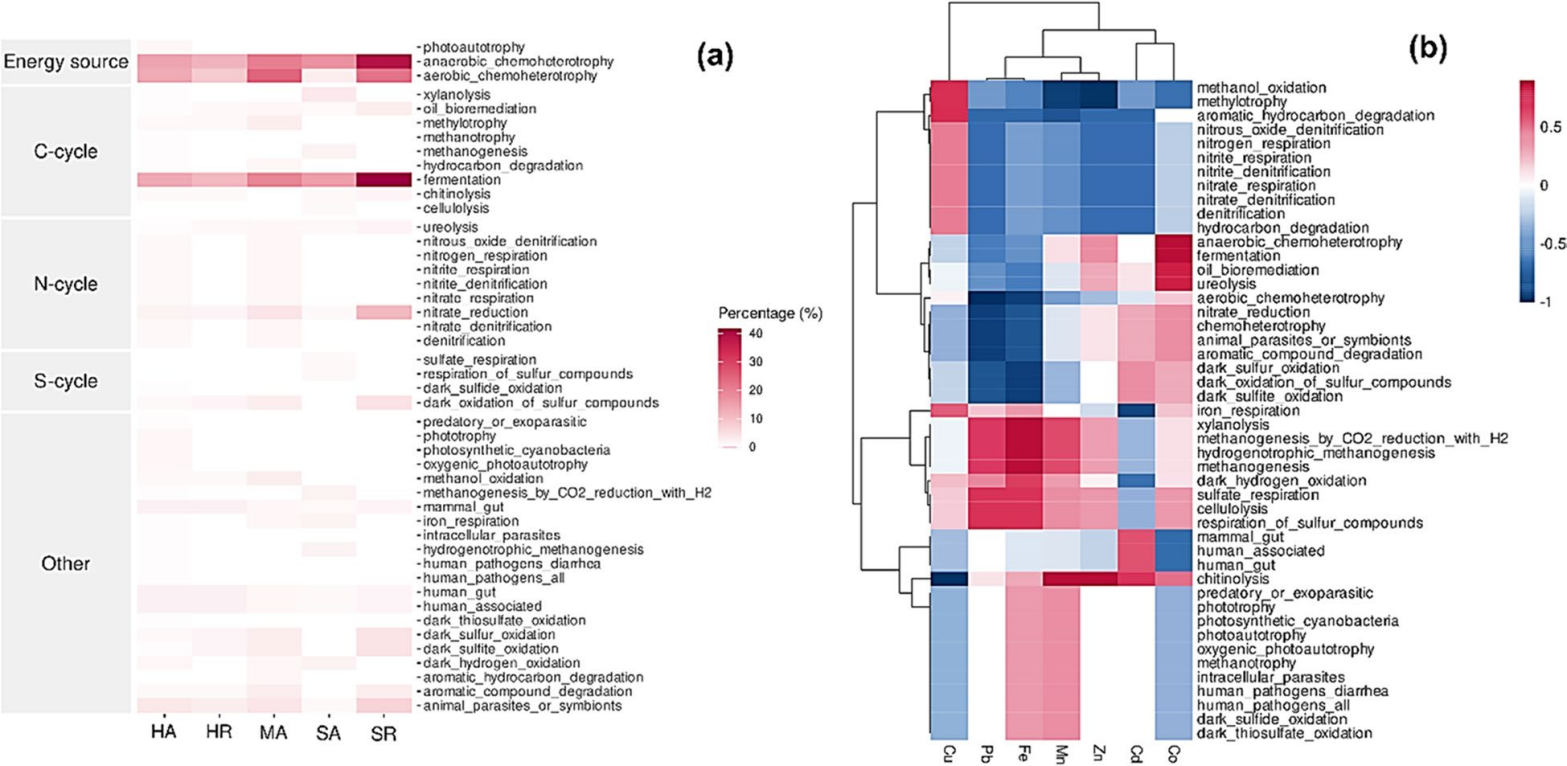
Heat-map visualization for **a** predicted functional pathways of 5 samples and **b** predicted functional correlation with heavy metals. Red colors indicate positive associations, whereas blue colors indicate negative associations. Heavy metals: Cu, copper; Pb, lead; Fe, iron; Mn, manganese; Zn, zinc; Cd, cadmium; and Co, cobalt. Locations and mangrove species: HA (Hamata, *Avicennia marina*), HR (Hamata, *Rhizophora mucronata*), MA (Mangrove Bay, *Avicennia marina*), SA (Saffaga, *Avicennia marina*), SR (Saffaga, *Rhizophora mucronata*)

Using the FAPROTAX database, microbial species were mapped to established metabolic pathways and other ecologically significant functions, and the results are summarized in Fig. 6a. Of the five clusters, cluster 1 (Energy source) and cluster 2 (Carbon cycle) showed a higher abundance of microbial species across all samples. Cluster 1 was composed of “anaerobic chemoheterotrophy” and “aerobic chemoheterotrophy,” whereas cluster 2 comprised “xylanolysis,” “oil bioremediation,” “methylotrophy,” “methanotrophy,” “methanogenesis,” “hydrocarbon degradation,” “fermentation,” “chitinolysis,” and “cellulolysis.” The microbial composition involved in anaerobic chemoheterotrophy and fermentation was higher in SR, followed by MA, HA, HR, and SA, respectively. Figure 6b presents the predicted functional correlation with HMs. Co showed strong and positive correlations with anaerobic chemoheterotrophy, fermentation, oil bioremediation, and ureolysis. Fe showed strong and positive correlations with xylanolysis, methanogenesis by carbon dioxide (CO_2_) reduction with hydrogen, hydrogenotrophic methanogenesis, and methanogenesis. Meanwhile, Mn and Zn showed a positive and strong correlation with chitinolysis.

**Fig. 7:**
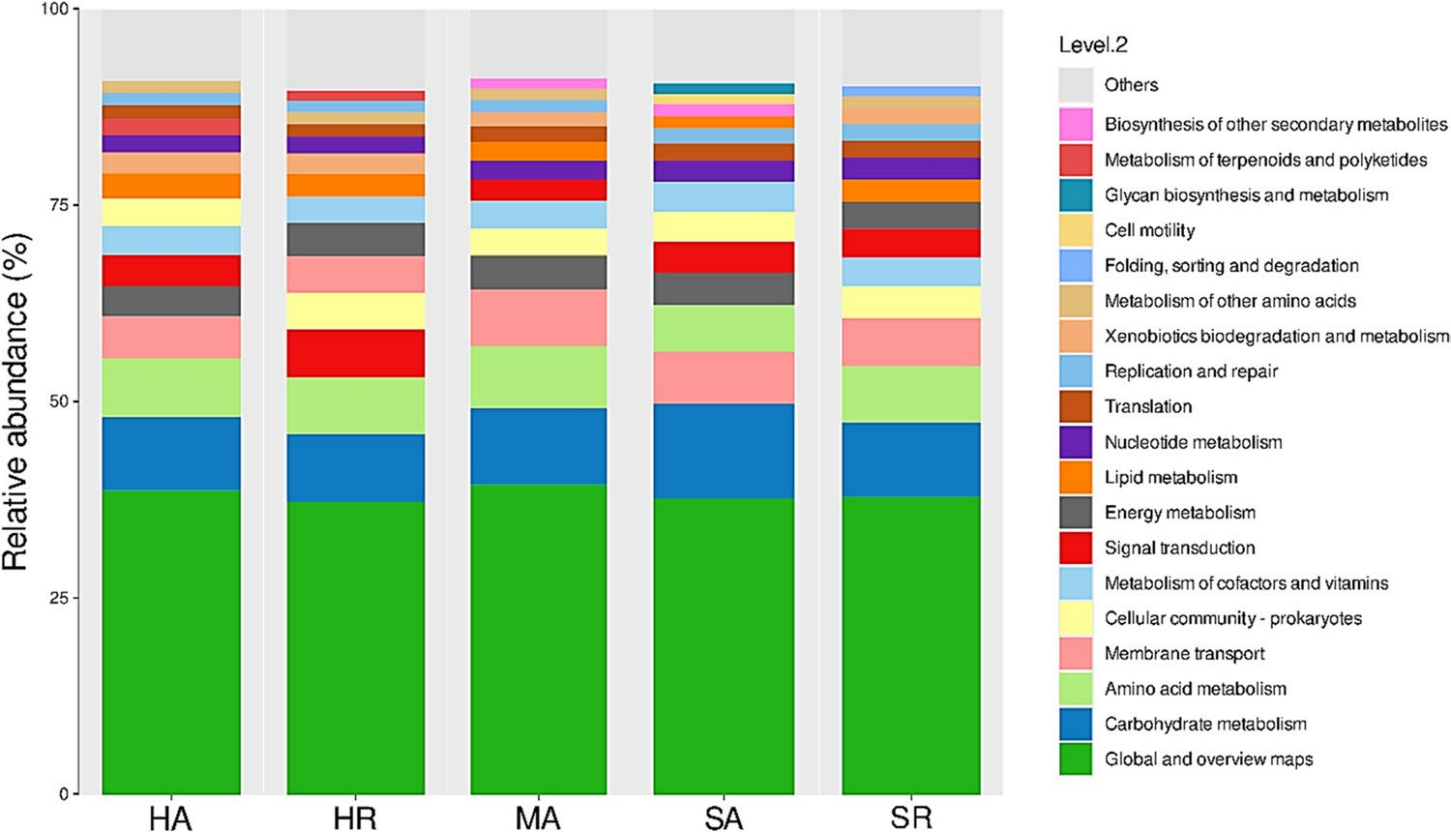
Functional prediction of unique operational taxonomic units found in sediment samples. Locations and mangrove species; HA (Hamata, *Avicennia marina*), HR (Hamata, *Rhizophora mucronata*), MA (Mangrove Bay, *Avicennia marina*), SA (Saffaga, *Avicennia marina*), SR (Saffaga, *Rhizophora mucronata*)

Tax4Fun2 database was used to predict the function of unique OTUs in each sediment sample and results are presented in Fig. 7. The common functions shared by the unique OTUs in sediment samples were global and overview maps, carbohydrate metabolism, amino acid metabolism, membrane transport, energy metabolism, signal transduction, metabolism of cofactors and vitamins, cellular community prokaryotes, lipid metabolism, nucleotide metabolism, translation, replication and repair. However, unique OTUs involved in xenobiotic biodegradation and repair and metabolism for other amino acids were abundant in most sediment samples except for SA. Unique OTUs involved in the metabolism of terpenoids and polyketides were only abundant in HA and HR sediment samples. Likewise, unique OTUs involved in the biosynthesis of other secondary metabolites were abundant in only the MA and SA sediment samples. SA sediment samples had a high abundance of unique OTUs involved in glycan biosynthesis and metabolism and cell motility. In the same regard, SR sediment samples had a high abundance of OTUs involved in folding, sorting, and degradation.

## Discussion

Microorganisms are crucial in maintaining the global biogeochemical cycling of nutrients in mangrove ecosystems (Aprilia et al. 2022; Meng et al. 2022; Yan et al. 2024). Their presence and activities are essential in the maintenance, conservation, and restoration of mangrove ecosystems (Holguin et al. 2001; Chen et al. 2016; Anu et al. 2024). In these ecosystems, bacteria and fungi are the most dominant microbes, comprising 91% of the total microbes present, followed by algae (7%) and protozoa (2%), respectively (Alongi et al. 1993; Nimnoi and Pongsilp 2022). However, microbial composition, diversity, and function in mangrove ecosystems are dependent on other environmental factors such as mangrove species, salinity, pH, electroconductivity (EC), anions and cations, elements, organic carbon (OC), and bulk density (BD).

In this study, the pH of the sediment samples ranged from 8.03 to 8.33 thus indicating the slight alkalinity of mangrove sediments along the Egyptian Red Sea coast at the mangrove sites of Hamata, Mangrove Bay, and Saffaga. This is consistent with previous studies on mangrove soils along the Red Sea coast of Saudi Arabia (Zhang et al. 2009; Sohaib et al. 2023), the Egyptian-African Red Sea coast (Afefe et al. 2019), and mangrove forest soil in Balandra Beach, Mexico (Gonzalez-acosta et al. 2006). However, slight acidity of mangrove soils has been reported in Ogle and Montrose (pH of 6.45 and 6.65) (Dookie et al. 2024), in a ‬‬‬‬‬‬‬‬‬‬‬‬‬‬‬‬‬‬‬‬‬‬‬‬‬‬‬‬‬‬‬‬‬‬‬‬‬‬‬‬‬‬‬‬‬‬‬‬‬‬‬‬‬‬‬‬‬‬‬‬‬‬‬‬‬‬‬‬‬‬‬‬‬‬‬‬‬‬‬‬‬‬‬‬‬‬‬‬‬‬‬‬‬‬‬‬‬‬‬‬‬‬‬‬‬‬‬‬‬‬‬‬‬‬‬‬‬‬‬‬‬‬‬‬‬‬‬‬‬‬‬‬‬‬‬‬‬‬‬‬‬‬‬‬‬‬‬‬‬‬‬‬‬‬‬‬‬‬‬‬‬‬‬‬‬‬‬‬‬‬‬‬‬‬‬‬‬‬‬‬‬‬‬‬‬‬‬‬‬‬‬‬‬‬‬‬‬‬‬‬‬‬‬‬‬‬‬‬‬‬‬‬‬‬‬‬‬‬‬‬‬‬‬‬‬‬‬‬‬‬‬‬‬‬‬‬‬‬‬‬‬‬‬‬‬‬‬‬‬‬‬‬‬‬‬‬‬‬‬‬‬‬‬‬‬‬‬‬‬‬‬‬‬‬‬‬‬‬‬‬‬‬‬‬‬‬‬‬‬‬‬‬‬‬‬‬‬‬‬‬mangrove swamp (pH of 5.30 to 6.80) in Nigeria (Ukpong 2007), and a non-mangrove rehabilitation site (pH of 5.90) in Indonesia (Dewiyanti et al. 2021). The best pH value for the successful growth of mangrove trees is approximately 6 to 8.50 since this pH range is favorable for aquatic life and microbial activity (Prihastanti et al. 2021). Electroconductivity (EC) indirectly influences the availability of nutrients for plant uptake as high and low EC values hinder and support nutrient absorption by plant roots respectively (do Carmo et al. 2024). The EC reported in this study ranged from 10.42 to 22.53 dS/m which is within the range of that reported in other studies along the Red Sea coastline (Abd Ellatif et al. 2023; Sohaib et al. 2023) but below that reported by Afefe et al. (2019). *A. marina* and *R. mucronata* species generally thrive in high and moderate salinity ranges of 5–35 ppt and 20–25 ppt, respectively, which translates to the EC value ranges of 8–55 dS/m for *A. marina* and 32–40 dS/m for *R. mucronata*. As such, the EC values reported in this study tend to favor *A. marina* more than they would favor *R. mucronata* and this could explain why *A marina* has thrived better than *R. mucronata* along the Egyptian Red Sea coastline. The bulk density (BD) of mangrove sediments ranged from 1.28 to 1.46 g/cm^3^ across the different depths, with significant differences observed at a 30–50 cm depth. Our study results are comparable to those of Eid and Shaltout (2016) who reported BD values of mangrove stands (*Avicennia marina*) along the Egyptian Red Sea coast to range from 1.00 to 1.50 g/cm^3^. The dense biomass of *A. marina* and *Rhizophora mucronata* root systems reduce sediment BD because of increasing biological activities that facilitate the conservation of some micropores into macropores due to the cementing action of polysaccharides and organic acids secreted during the decomposition of organic matter (OM) by microorganisms (Sombrero and Benito 2010; Eid and Shaltout 2016). On the other hand, the sediment organic carbon (OC) percentage was higher in the upper depths (0–15 cm and 15–30 cm) than in lower depths (50–100 cm) for HA (Hamata; *A. marina*) and HR (Hamata; *R. mucronata*) sediment samples which is consistent with previous studies (Islam and Rempei 2007; Eid and Shaltout 2013; Wang et al. 2013; Lunstrum and Chen 2014; Yang et al. 2014). Most carbon inputs occur in the upper soil surface. Moreover, the upper soil surface (0–10 cm) accounts for approximately 35% of the mean total OC content which confirms its significant role in the carbon cycle (i.e., carbon sink and release of carbon dioxide) (Eid and Shaltout 2016; Saini et al. 2021; Brahim et al. 2022). However, our study results also show variations in the OC percentage in sediment samples of MA (Mangrove Bay; *A. marina*), SA (Saffaga; *A. marina*), and SR (Saffaga; *R. mucronata*) at different depths. This variation is attributed to several factors such as leaching, soil erosion, nutrient cycling, soil illuviation, mineral weathering, and decomposition (Girmay and Singh 2012).‬‬‬‬‬‬‬‬‬‬‬‬‬‬‬‬‬‬‬‬‬‬‬‬‬‬‬‬‬‬‬‬‬‬‬‬‬‬‬‬‬‬‬‬‬‬‬‬‬‬‬‬‬‬‬‬‬‬‬‬‬‬‬‬‬‬‬‬‬‬‬‬‬‬‬‬‬‬‬‬‬‬‬‬‬‬‬‬‬‬‬‬‬‬‬‬‬‬‬‬‬‬‬‬‬‬‬‬‬‬‬‬‬‬‬‬‬‬‬‬‬‬‬‬‬‬‬‬‬‬‬‬‬‬‬‬‬‬‬‬‬‬‬‬‬‬‬‬‬‬‬‬‬‬‬‬‬‬‬‬‬‬‬‬‬‬‬‬‬‬‬‬‬‬‬‬‬‬‬‬‬‬‬‬‬‬‬‬‬‬‬‬‬‬‬‬‬‬‬‬‬‬‬‬‬‬‬‬‬‬‬‬‬‬‬‬‬‬‬‬‬‬‬‬‬‬‬‬‬‬‬‬‬‬‬‬‬‬‬‬‬‬‬‬‬‬‬‬‬‬‬‬‬‬‬‬‬‬‬‬‬‬‬‬‬‬‬‬‬‬‬‬‬‬‬‬‬‬‬‬‬‬‬‬‬‬‬‬‬‬‬‬‬‬‬‬‬‬‬‬‬‬‬‬‬‬‬‬‬‬‬‬‬‬‬‬‬‬‬‬‬‬‬‬‬‬‬‬‬‬‬‬‬‬‬‬‬‬‬‬‬‬‬‬‬‬‬‬‬‬‬‬‬‬‬‬‬‬‬‬‬‬‬‬‬‬‬‬‬‬‬‬‬‬‬‬‬‬‬‬‬‬‬‬‬‬‬‬‬‬‬‬‬‬‬‬‬‬‬‬‬‬‬‬‬‬‬‬‬‬‬‬‬‬‬‬‬‬‬‬‬‬‬‬‬‬‬‬‬‬‬‬‬‬‬‬‬‬‬‬‬‬‬‬‬‬‬‬‬‬‬‬‬‬‬‬‬‬‬‬‬‬‬‬‬‬‬‬‬‬‬‬‬‬‬‬‬‬‬‬‬‬‬‬‬‬‬‬‬‬‬‬‬‬‬‬‬‬‬‬‬‬‬‬‬‬‬‬‬‬‬‬‬‬‬‬‬‬‬‬‬‬‬‬‬‬‬‬‬‬‬‬‬‬‬‬‬‬‬‬‬‬‬‬‬‬‬‬‬‬‬‬‬‬‬‬‬‬‬‬‬‬‬‬‬‬‬‬‬‬‬‬‬‬‬‬‬‬‬‬‬‬‬‬‬‬‬‬‬‬‬‬‬‬‬‬‬‬‬‬‬‬‬‬‬‬‬‬‬‬‬‬‬‬‬‬‬‬‬‬‬‬‬‬‬‬‬‬‬‬‬‬‬‬‬‬‬‬‬‬‬‬‬‬‬‬‬‬‬‬‬‬‬‬‬‬‬‬‬‬‬‬‬‬‬‬‬‬‬‬‬‬‬‬‬‬‬‬‬‬‬‬‬‬‬‬‬‬‬‬‬‬‬‬‬‬‬‬‬‬‬‬‬‬‬‬‬‬‬‬‬‬‬‬‬‬‬‬‬‬‬‬‬‬‬‬‬‬‬‬‬‬‬‬‬‬‬‬‬‬‬‬‬‬‬‬‬‬‬‬‬‬‬‬‬‬‬‬‬‬‬‬‬‬‬‬‬‬‬‬‬‬‬‬‬‬‬‬‬‬‬‬‬‬‬‬‬‬‬‬‬‬‬‬‬‬‬‬‬‬‬‬‬‬‬‬‬‬‬‬‬‬‬‬‬‬‬‬‬‬‬‬‬‬‬‬‬‬‬‬‬‬‬‬‬‬‬‬‬‬‬‬‬‬‬‬‬‬‬‬‬‬‬‬‬‬‬‬‬‬‬‬‬‬‬‬‬‬‬‬‬‬‬‬‬‬‬‬‬‬‬‬‬‬‬‬‬‬‬‬‬‬‬‬‬‬‬‬‬‬‬‬‬‬‬‬‬‬‬‬‬‬‬‬‬‬‬‬‬‬‬‬‬‬‬‬‬‬‬‬‬‬‬‬‬‬‬‬‬‬‬‬‬‬‬‬‬‬‬‬‬‬‬‬‬‬‬‬‬‬‬‬‬‬‬‬‬‬‬‬‬‬‬‬‬‬‬‬‬‬‬‬‬‬‬‬‬‬‬‬‬‬‬‬‬‬‬‬‬‬‬‬‬‬‬‬‬‬‬‬‬‬‬‬‬‬‬‬‬‬‬‬‬‬‬‬‬‬‬‬‬‬‬‬‬‬‬‬‬‬‬‬‬‬‬‬‬‬‬‬‬‬‬‬‬‬‬‬‬‬‬‬‬‬‬‬‬‬‬‬‬‬‬‬‬‬‬‬‬‬‬‬‬‬‬‬‬‬‬‬‬‬‬‬‬‬‬‬‬‬‬‬‬‬‬‬‬‬‬‬‬‬‬‬‬‬‬‬‬‬‬‬‬‬‬‬‬‬‬‬‬‬‬‬‬‬‬‬‬‬‬‬‬‬‬‬‬‬‬‬‬‬‬‬‬‬‬‬‬‬‬‬‬‬‬‬‬‬‬‬‬‬‬‬‬‬‬‬‬‬‬‬‬‬‬‬‬‬‬‬‬‬‬‬‬‬‬‬‬‬‬‬‬‬‬‬‬‬‬‬‬‬‬‬‬‬‬‬‬‬‬‬‬‬‬‬‬‬‬‬‬‬‬‬‬‬‬‬‬‬‬‬‬‬‬‬‬‬‬‬‬‬‬‬‬‬‬‬‬‬‬‬‬‬‬‬‬‬‬‬‬‬‬‬‬‬‬‬‬‬‬‬‬‬‬‬‬‬‬‬‬‬‬‬‬‬‬‬‬‬‬‬‬‬‬‬‬‬‬‬‬‬‬‬‬‬‬‬‬‬‬‬‬‬‬‬‬‬‬‬‬‬‬‬‬‬‬‬‬‬‬‬‬‬‬‬‬‬‬‬‬‬‬‬‬‬‬‬‬‬‬‬‬‬‬‬‬‬‬‬‬‬‬‬‬‬‬‬‬‬‬‬‬‬‬‬‬‬‬‬‬‬‬‬‬‬‬‬‬‬‬‬‬‬‬‬‬‬‬‬‬‬‬‬‬‬‬‬‬‬‬‬‬‬‬‬‬‬‬‬‬‬‬‬‬‬‬‬‬‬‬‬‬‬‬‬‬‬‬‬‬‬‬‬‬‬‬‬‬‬‬‬‬‬‬‬‬‬‬‬‬‬‬‬‬‬‬‬‬‬‬‬‬‬‬‬‬‬‬‬‬‬‬‬‬‬‬‬‬‬‬‬‬‬‬‬‬‬‬‬‬‬‬‬‬‬‬‬‬‬‬‬‬‬‬‬‬‬‬‬‬‬‬‬‬‬‬‬‬‬‬‬‬‬‬‬‬‬‬‬‬‬‬‬‬‬‬‬‬‬‬‬‬‬‬‬‬‬‬‬‬‬‬‬‬‬‬‬‬‬‬‬‬‬‬‬‬‬‬‬‬‬‬‬‬‬‬‬‬‬‬‬‬‬‬‬‬‬‬‬‬‬‬‬‬‬‬‬‬‬‬‬‬‬‬‬‬‬‬‬‬‬‬‬‬‬‬‬‬‬‬‬‬‬‬‬‬‬‬‬‬‬‬‬‬‬‬‬‬‬‬‬‬‬‬‬‬‬‬‬‬‬‬‬‬‬‬‬‬‬‬‬‬‬‬‬‬‬‬‬‬‬‬‬‬‬‬‬‬‬‬‬‬‬‬‬‬‬‬‬‬‬‬‬‬‬‬‬‬‬‬‬‬‬‬‬‬‬‬‬‬‬‬‬‬‬‬‬‬‬‬‬‬‬‬‬‬‬‬‬‬‬‬‬‬‬‬‬‬‬‬‬‬‬‬‬‬‬‬‬‬‬‬‬‬‬‬‬‬‬‬‬‬‬‬‬‬‬‬‬‬‬‬‬‬‬‬‬‬‬‬‬‬‬‬‬‬‬‬‬‬‬‬‬‬‬‬‬‬‬‬‬‬‬‬‬‬‬‬‬‬‬‬‬‬‬‬‬‬‬‬‬‬‬‬‬‬‬‬‬‬‬‬‬‬‬‬‬‬‬‬‬‬‬‬‬‬‬‬‬‬‬‬‬‬‬‬‬‬‬‬‬‬‬‬‬‬‬‬‬‬‬‬‬‬‬‬‬‬‬‬‬‬‬‬‬‬‬‬‬‬‬‬‬‬‬‬‬‬‬‬‬‬‬‬‬‬‬‬‬‬‬‬‬‬‬‬‬‬‬‬‬‬‬‬‬‬‬‬‬‬‬‬‬‬‬‬‬‬‬‬‬‬‬‬‬‬‬‬‬‬‬‬‬‬‬‬‬‬‬‬‬‬‬‬‬‬‬‬‬‬‬‬‬‬‬‬‬‬‬‬‬‬‬‬‬‬‬‬‬‬‬‬‬‬‬‬‬‬‬‬‬‬‬‬‬‬‬‬‬‬‬‬‬‬‬‬‬‬‬‬‬‬‬‬‬‬‬‬‬‬‬‬‬‬‬‬‬‬‬‬‬‬‬‬‬‬‬‬‬‬‬‬‬‬‬‬‬‬‬‬‬‬‬‬‬‬‬‬‬‬‬‬‬‬‬‬‬‬‬‬‬‬‬‬‬‬‬‬‬‬‬‬‬‬‬‬‬‬‬‬‬‬‬‬‬‬‬‬‬‬‬‬‬‬‬‬‬‬‬‬‬‬‬‬‬‬‬‬‬‬‬‬‬‬‬‬‬‬‬‬‬‬‬‬‬‬‬‬‬‬‬‬‬‬‬‬‬‬‬‬‬‬‬‬‬‬‬‬‬‬‬‬‬‬‬‬‬‬‬‬‬‬‬‬‬‬‬‬‬‬‬‬‬‬‬‬‬‬‬‬‬‬‬‬‬‬‬‬‬‬‬‬‬‬‬‬‬‬‬‬‬‬‬‬‬‬‬‬‬‬‬‬‬‬‬‬‬‬‬‬‬‬‬‬‬‬‬‬‬‬‬‬‬‬‬‬‬‬‬‬‬‬‬‬‬‬‬‬‬‬‬‬‬‬‬‬‬‬‬‬‬‬‬‬‬‬‬‬‬‬‬‬‬‬‬‬‬‬‬‬‬‬‬‬‬‬‬‬‬‬‬‬‬‬‬‬‬‬‬‬‬‬‬‬‬‬‬‬‬‬‬‬‬‬‬‬‬‬‬‬‬‬‬‬‬‬‬‬‬‬‬‬‬‬‬‬‬‬‬‬‬‬‬‬‬‬‬‬‬‬‬‬‬‬‬‬‬‬‬‬‬‬‬‬‬‬‬‬‬‬‬‬‬‬‬‬‬‬‬‬‬‬‬‬‬‬‬‬‬‬‬‬‬‬‬‬‬‬‬‬‬‬‬‬‬‬‬‬‬‬‬‬‬‬‬‬‬‬‬‬‬‬‬‬‬‬‬‬‬‬‬‬‬‬‬‬‬‬‬‬‬‬‬‬‬‬‬‬‬‬‬‬‬‬‬‬‬‬‬‬‬‬‬‬‬‬‬‬‬‬‬‬‬‬‬‬‬‬‬‬‬‬‬‬‬‬‬‬‬‬‬‬‬‬‬‬‬‬‬‬‬‬‬‬‬‬‬‬‬‬‬‬‬‬‬‬‬‬‬‬‬‬‬‬‬‬‬‬‬‬‬‬‬‬‬‬‬‬‬‬‬‬‬‬‬‬‬‬‬‬‬‬‬‬‬‬‬‬‬‬‬‬‬‬‬‬‬‬‬‬‬‬‬‬‬‬‬‬‬‬‬‬‬‬‬‬‬‬‬‬‬‬‬‬‬‬‬‬‬‬‬‬‬‬‬‬‬‬‬‬‬‬‬‬‬‬‬‬‬‬‬‬‬‬‬‬‬‬‬‬‬‬‬‬‬‬‬‬‬‬‬‬‬‬‬‬‬‬‬‬‬‬‬‬‬‬‬‬‬‬‬‬‬‬‬‬‬‬‬‬‬‬‬‬‬‬‬‬‬‬‬‬‬‬‬‬‬‬‬‬‬‬‬‬‬‬‬‬‬‬‬‬‬‬‬‬‬‬‬‬‬‬‬‬‬‬‬‬‬‬‬‬‬‬‬‬‬‬‬‬‬‬‬‬‬‬‬‬‬‬‬‬‬‬‬‬‬‬‬‬‬‬‬‬‬‬‬‬‬‬‬‬‬‬‬‬‬‬‬‬‬‬‬‬‬‬‬‬‬‬‬‬‬‬‬‬‬‬‬‬‬‬‬‬‬‬‬‬‬‬‬‬‬‬‬‬‬‬‬‬‬‬‬‬‬‬‬‬‬‬‬‬‬‬‬‬‬‬‬‬‬‬‬‬‬‬‬‬‬‬‬‬‬‬‬‬‬‬‬‬‬‬‬‬‬‬‬‬‬‬‬‬‬‬‬‬‬‬‬‬‬‬‬‬‬‬‬‬‬‬‬‬‬‬‬‬‬‬‬‬‬‬‬‬‬‬‬‬‬‬‬‬‬‬‬‬‬‬‬‬‬‬‬‬‬‬‬‬‬‬‬‬‬‬‬‬‬‬‬‬‬‬‬‬‬‬‬‬‬‬‬‬‬‬‬‬‬‬‬‬‬‬‬‬‬‬‬‬‬‬‬‬‬‬‬‬‬‬‬‬‬‬‬‬‬‬‬‬‬‬‬‬‬‬‬‬‬‬‬‬‬‬‬‬‬‬‬‬‬‬‬‬‬‬‬‬‬‬‬‬‬‬‬‬‬‬‬‬‬‬‬‬‬‬‬‬‬‬‬‬‬‬‬‬‬‬‬‬‬‬‬‬‬‬‬‬‬‬‬‬‬‬‬‬‬‬‬‬‬‬‬‬‬‬‬‬‬‬‬‬‬‬‬‬‬‬‬‬‬‬‬‬‬‬‬‬‬‬‬‬‬‬‬‬‬‬‬‬‬‬‬‬‬‬‬‬‬‬‬‬‬‬‬‬‬‬‬‬‬‬‬‬‬‬‬‬‬‬‬‬‬‬‬‬‬‬‬‬‬‬‬‬‬‬‬‬‬‬‬‬‬‬‬‬‬‬‬‬‬‬‬‬‬‬‬‬‬‬‬‬‬‬‬‬‬‬‬‬‬‬‬‬‬‬‬‬‬‬‬‬‬‬‬‬‬‬‬‬‬‬‬‬‬‬‬‬‬‬‬‬‬‬‬‬‬‬‬‬‬‬‬‬‬‬‬‬‬‬‬‬‬‬‬‬‬‬‬‬‬‬‬‬‬‬‬‬‬‬‬‬‬‬‬‬‬‬‬‬‬‬‬‬‬‬‬‬‬‬‬‬‬‬‬‬‬‬‬‬‬‬‬‬‬‬‬‬‬‬‬‬‬‬‬‬‬‬‬‬‬‬‬‬‬‬‬‬‬‬‬‬‬‬‬‬‬‬‬‬‬‬‬‬‬‬‬‬‬‬‬‬‬‬‬‬‬‬‬‬‬‬‬‬‬‬‬‬‬‬‬‬‬‬‬‬‬‬‬‬‬‬‬‬‬‬‬‬‬‬‬‬‬‬‬‬‬‬‬‬‬‬‬‬‬‬‬‬‬‬‬‬‬‬‬‬‬‬‬‬‬‬‬‬‬‬‬‬‬‬‬‬‬‬‬‬‬‬‬‬‬‬‬‬‬‬‬‬‬‬‬‬‬‬‬‬‬‬‬‬‬‬‬‬‬‬‬‬‬‬‬‬‬‬‬‬‬‬‬‬‬‬‬‬‬‬‬‬‬‬‬‬‬‬‬‬‬‬‬‬‬‬‬‬‬‬‬‬‬‬‬‬‬‬‬‬‬‬‬‬‬‬‬‬‬‬‬‬‬‬‬‬‬‬‬‬‬‬‬‬‬‬‬‬‬‬‬‬‬‬‬‬‬‬‬‬‬‬‬‬‬‬‬‬‬‬‬‬‬‬‬‬‬‬‬‬‬‬‬‬‬‬‬‬‬‬‬‬‬‬‬‬‬‬‬‬‬‬‬‬‬‬‬‬‬‬‬‬‬‬‬‬‬‬‬‬‬‬‬‬‬‬‬‬‬‬‬‬‬‬‬‬‬‬‬‬‬‬‬‬‬‬‬‬‬‬‬‬‬‬‬‬‬‬‬‬‬‬‬‬‬‬‬‬‬‬‬‬‬‬‬‬‬‬‬‬‬‬‬‬‬‬‬‬‬‬‬‬‬‬‬‬‬‬‬‬‬‬‬‬‬‬‬‬‬‬‬‬‬‬‬‬‬‬‬‬‬‬‬‬‬‬‬‬‬‬‬‬‬‬‬‬‬‬‬‬‬‬‬‬‬‬‬‬‬‬‬‬‬‬‬‬‬‬‬‬‬‬‬‬‬‬‬‬‬‬‬‬‬‬‬‬‬‬‬‬‬‬‬‬‬‬‬‬‬‬‬‬‬‬‬‬‬‬‬‬‬‬‬‬‬‬‬‬‬‬‬‬‬‬‬‬‬‬‬‬‬‬‬‬‬‬‬‬‬‬‬‬‬‬‬‬‬‬‬‬‬‬‬‬‬‬‬‬‬‬‬‬‬‬‬‬‬‬‬‬‬‬‬‬‬‬‬‬‬‬‬‬‬‬‬‬‬‬‬‬‬‬‬‬‬‬‬‬‬‬‬‬‬‬‬‬‬‬‬‬‬‬‬‬‬‬‬‬‬‬‬‬‬‬‬‬‬‬‬‬‬‬‬‬‬‬‬‬‬‬‬‬‬‬‬‬‬‬‬‬‬‬‬‬‬‬‬‬‬‬‬‬‬‬‬‬‬‬‬‬‬‬‬‬‬‬‬‬‬‬‬‬‬‬‬‬‬‬‬‬‬‬‬‬‬‬‬‬‬‬‬‬‬‬‬‬‬‬‬‬‬‬‬‬‬‬‬‬‬‬‬‬‬‬‬‬‬‬‬‬‬‬‬‬‬‬‬‬‬‬‬‬‬‬‬‬‬‬‬‬‬‬‬‬‬‬‬‬‬‬‬‬‬‬‬‬‬‬‬‬‬‬‬‬‬‬‬‬‬‬‬‬‬‬‬‬‬‬‬‬‬‬‬‬‬‬‬‬‬‬‬‬‬‬‬‬‬‬‬‬‬‬‬‬‬‬‬‬‬‬‬‬‬‬‬‬‬‬‬‬‬‬‬‬‬‬‬‬‬‬‬‬‬‬‬‬‬‬‬‬‬‬‬‬‬‬‬‬‬‬‬‬‬‬‬‬‬‬‬‬‬‬‬‬‬‬‬‬‬‬‬‬‬‬‬‬‬‬‬‬‬‬‬‬‬‬‬‬‬‬‬‬‬‬‬‬‬‬‬‬‬‬‬‬‬‬‬‬‬‬‬‬‬‬‬‬‬‬‬‬‬‬‬‬‬‬‬‬‬‬‬‬‬‬‬‬‬‬‬‬‬‬‬‬‬‬‬‬‬‬‬‬‬‬‬‬‬‬‬‬‬‬‬‬‬‬‬‬‬‬‬‬‬‬‬‬‬‬‬‬‬‬‬‬‬‬‬‬‬‬‬‬‬‬‬‬‬‬‬‬‬‬‬‬‬‬‬‬‬‬‬‬‬‬‬‬‬‬‬‬‬‬‬‬‬‬‬‬‬‬‬‬‬‬‬‬‬‬‬‬‬‬‬‬‬‬‬‬‬‬‬‬‬‬‬‬‬‬‬‬‬‬‬‬‬‬‬‬‬‬‬‬‬‬‬‬‬‬‬‬‬‬‬‬‬‬‬‬‬‬‬‬‬‬‬‬‬‬‬‬‬‬‬‬‬‬‬‬‬‬‬‬‬‬‬‬‬‬‬‬‬‬‬‬‬‬‬‬‬‬‬‬‬‬‬‬‬‬‬‬‬‬‬‬‬‬‬‬‬‬‬‬‬‬‬‬‬‬‬‬‬‬‬‬‬‬‬‬‬‬‬‬‬‬‬‬‬‬‬‬‬‬‬‬‬‬‬‬‬‬‬‬‬‬‬‬‬‬‬‬‬‬‬‬‬‬‬‬‬‬‬‬‬‬‬‬‬‬‬‬‬‬‬‬‬‬‬‬‬‬‬‬‬‬‬‬‬‬‬‬‬‬‬‬‬‬‬‬‬‬‬‬‬‬‬‬‬‬‬‬‬‬‬‬‬‬‬‬‬‬‬‬‬‬‬‬‬‬‬‬‬‬‬‬‬‬‬‬‬‬‬‬‬‬‬‬‬‬‬‬‬‬‬‬‬‬‬‬‬‬‬‬‬‬‬‬‬‬‬‬‬‬‬‬‬‬‬‬‬‬‬‬‬‬‬‬‬‬‬‬‬‬‬‬‬‬‬‬‬‬‬‬‬‬‬‬‬‬‬‬‬‬‬‬‬‬‬‬‬‬‬‬‬‬‬‬‬‬‬‬‬‬‬‬‬‬‬‬‬‬‬‬‬‬‬‬‬‬‬‬‬‬‬‬‬‬‬‬‬‬‬‬‬‬‬‬‬‬‬‬‬‬‬‬‬‬‬‬‬‬‬‬‬‬‬‬‬‬‬‬‬‬‬‬‬‬‬‬‬‬‬‬‬‬‬‬‬‬‬‬‬‬‬‬‬‬‬‬‬‬‬‬‬‬‬‬‬‬‬‬‬‬‬‬‬‬‬‬‬‬‬‬‬‬‬‬‬‬‬‬‬‬‬‬‬‬‬‬‬‬‬‬‬‬‬‬‬‬‬‬‬‬‬‬‬‬‬‬‬‬‬‬‬‬‬‬‬‬‬‬‬‬‬‬‬‬‬‬‬‬‬‬‬‬‬‬‬‬‬‬‬‬‬‬‬‬‬‬‬‬‬‬‬‬‬‬‬‬‬‬‬‬‬‬‬‬‬‬‬‬‬‬‬‬‬‬‬‬‬‬‬‬‬‬‬‬‬‬‬‬‬‬‬‬‬‬‬‬‬‬‬‬‬‬‬‬‬‬‬‬‬‬‬‬‬‬‬‬‬‬‬‬‬‬‬‬‬‬‬‬‬‬‬‬‬‬‬‬‬‬‬‬‬‬‬‬‬‬‬‬‬‬‬‬‬‬‬‬‬‬‬‬‬‬‬‬‬‬‬‬‬‬‬‬‬‬‬‬‬‬‬‬‬‬‬‬‬‬‬‬‬‬‬‬‬‬‬‬‬‬‬‬‬‬‬‬‬‬‬‬‬‬‬‬‬‬‬‬‬‬‬‬‬‬‬‬‬‬‬‬‬‬‬‬‬‬‬‬‬‬‬‬‬‬‬‬‬‬‬‬‬‬‬‬‬‬‬‬‬‬‬‬‬‬‬‬‬‬‬‬‬‬‬‬‬‬‬‬‬‬‬‬‬‬‬‬‬‬‬‬‬‬‬‬‬‬‬‬‬‬‬‬‬‬‬‬‬‬‬‬‬‬‬‬‬‬‬‬‬‬‬‬‬‬‬‬‬‬‬‬‬‬‬‬‬‬‬‬‬‬‬‬‬‬‬‬‬‬‬‬‬‬‬‬‬‬‬‬‬‬‬‬‬‬‬‬‬‬‬‬‬‬‬‬‬‬‬‬‬‬‬‬‬‬‬‬‬‬‬‬‬‬‬‬‬‬‬‬‬‬‬‬‬‬‬‬‬‬‬‬‬‬‬‬‬‬‬‬‬‬‬‬‬‬‬‬‬‬‬‬‬‬‬‬‬‬‬‬‬‬‬‬‬‬‬‬‬‬‬‬‬‬‬‬‬‬‬‬‬‬‬‬‬‬‬‬‬‬‬‬‬‬‬‬‬‬‬‬‬‬‬‬‬‬‬‬‬‬‬‬‬‬‬‬‬‬‬‬‬‬‬‬‬‬‬‬‬‬‬‬‬‬‬‬‬‬‬‬‬‬‬‬‬‬‬‬‬‬‬‬‬‬‬‬‬‬‬‬‬‬‬‬‬‬‬‬‬‬‬‬‬‬‬‬‬‬‬‬‬‬‬‬‬‬‬‬‬‬‬‬‬‬‬‬‬‬‬‬‬‬‬‬‬‬‬‬‬‬‬‬‬‬‬‬‬‬‬‬‬‬‬‬‬‬‬‬‬‬‬‬‬‬‬‬‬‬‬‬‬‬‬‬‬‬‬‬‬‬‬‬‬‬‬‬‬‬‬‬‬‬‬‬‬‬‬‬‬‬‬‬‬‬‬‬‬‬‬‬‬‬‬‬‬‬‬‬‬‬‬‬‬‬‬‬‬‬‬‬‬‬‬‬‬‬‬‬‬‬‬‬‬‬‬‬‬‬‬‬‬‬‬‬‬‬‬‬‬‬‬‬‬‬‬‬‬‬‬‬‬‬‬‬‬‬‬‬‬‬‬‬‬‬‬‬‬‬‬‬‬‬‬‬‬‬‬‬‬‬‬‬‬‬‬‬‬‬‬‬‬‬‬‬‬‬‬‬‬‬‬‬‬‬‬‬‬‬‬‬‬‬‬‬‬‬‬‬‬‬‬‬‬‬‬‬‬‬‬‬‬‬‬‬‬‬‬‬‬‬‬‬‬‬‬‬‬‬‬‬‬‬‬‬‬‬‬‬‬‬‬‬‬‬‬‬‬‬‬‬‬‬‬‬‬‬‬‬‬‬‬‬‬‬‬‬‬‬‬‬‬‬‬‬‬‬‬‬‬‬‬‬‬‬‬‬‬‬‬‬‬‬‬‬‬‬‬‬‬‬‬‬‬‬‬‬‬‬‬‬‬‬‬‬‬‬‬‬‬‬‬‬‬‬‬‬‬‬‬‬‬‬‬‬‬‬‬‬‬‬‬‬‬‬‬‬‬‬‬‬‬‬‬‬‬‬‬‬‬‬‬‬‬‬‬‬‬‬‬‬‬‬‬‬‬‬‬‬‬‬‬‬‬‬‬‬‬‬‬‬‬‬‬‬‬‬‬‬‬‬‬‬‬‬‬‬‬‬‬‬‬‬‬‬‬‬‬‬‬‬‬‬‬‬‬‬‬‬‬‬‬‬‬‬‬‬‬‬‬‬‬‬‬‬‬‬‬‬‬‬‬‬‬‬‬‬‬‬‬‬‬‬‬‬‬‬‬‬‬‬‬‬‬‬‬‬‬‬‬‬‬‬‬‬‬‬‬‬‬‬‬‬‬‬‬‬‬‬‬‬‬‬‬‬‬‬‬‬‬‬‬‬‬‬‬‬‬‬‬‬‬‬‬‬‬‬‬‬‬‬‬‬‬‬‬‬‬‬‬‬‬‬‬‬‬‬‬‬‬‬‬‬‬‬‬‬‬‬‬‬‬‬‬‬‬‬‬‬‬‬‬‬‬‬‬‬‬‬‬‬‬‬‬‬‬‬‬‬‬‬‬‬‬‬‬‬‬‬‬‬‬‬‬‬‬‬‬‬‬‬‬‬‬‬‬‬‬‬‬‬‬‬‬‬‬‬‬‬‬‬‬‬‬‬‬‬‬‬‬‬‬‬‬‬‬‬‬‬‬‬‬‬‬‬‬‬‬‬‬‬‬‬‬‬‬‬‬‬‬‬‬‬‬‬‬‬‬‬‬‬‬‬‬‬‬‬‬‬‬‬‬‬‬‬‬‬‬‬‬‬‬‬‬‬‬‬‬‬‬‬‬‬‬‬‬‬‬‬‬‬‬‬‬‬‬‬‬‬‬‬‬‬‬‬‬‬‬‬‬‬‬‬‬‬‬‬‬‬‬‬‬‬‬‬‬‬‬‬‬‬‬‬‬‬‬‬‬‬‬‬‬‬‬‬‬‬‬‬‬‬‬‬‬‬‬‬‬‬‬‬‬‬‬‬‬‬‬‬‬‬‬‬‬‬‬‬‬‬‬‬‬‬‬‬‬‬‬‬‬‬‬‬‬‬‬‬‬‬‬‬‬‬‬‬‬‬‬‬‬‬‬‬‬‬‬‬‬‬‬‬‬‬‬‬‬‬‬‬‬‬‬‬‬‬‬‬‬‬‬‬‬‬‬‬‬‬‬‬‬‬‬‬‬‬‬‬‬‬‬‬‬‬‬‬‬‬‬‬‬‬‬‬‬‬‬‬‬‬‬‬‬‬‬‬‬‬‬‬‬‬‬‬‬‬‬‬‬‬‬‬‬‬‬‬‬‬‬‬‬‬‬‬‬‬‬‬‬‬‬‬‬‬‬‬‬‬‬‬‬‬‬‬‬‬‬‬‬‬‬‬‬‬‬‬‬‬‬‬‬‬‬‬‬‬‬‬‬‬‬‬‬‬‬‬‬‬‬‬‬‬‬‬‬‬‬‬‬‬‬‬‬‬‬‬‬‬‬‬‬‬‬‬‬‬‬‬‬‬‬‬‬‬‬‬‬‬‬‬‬‬‬‬‬‬‬‬‬‬‬‬‬‬‬‬‬‬‬‬‬‬‬‬‬‬‬‬‬‬‬‬‬‬‬‬‬‬‬‬‬‬‬‬‬‬‬‬‬‬‬‬‬‬‬‬‬‬‬‬‬‬‬‬‬‬‬‬‬‬‬‬‬‬‬‬‬‬‬‬‬‬‬‬‬‬‬‬‬‬‬‬‬‬‬‬‬‬‬‬‬‬‬‬‬‬‬‬‬‬‬‬‬‬‬‬‬‬‬‬‬‬‬‬‬‬‬‬‬‬‬‬‬‬‬‬‬‬‬‬‬‬‬‬‬‬‬‬‬‬‬‬‬‬‬‬‬‬‬‬‬‬‬‬‬‬‬‬‬‬‬‬‬‬‬‬‬‬‬‬‬‬‬‬‬‬‬‬‬‬‬‬‬‬‬‬‬‬‬‬‬‬‬‬‬‬‬‬‬‬‬‬‬‬‬‬‬‬‬‬‬‬‬‬‬‬‬‬‬‬‬‬‬‬‬‬‬‬‬‬‬‬‬‬‬‬‬‬‬‬‬‬‬‬‬‬‬‬‬‬‬‬‬‬‬‬‬‬‬‬‬‬‬‬‬‬‬‬‬‬‬‬‬‬‬‬‬‬‬‬‬‬‬‬‬‬‬‬‬‬‬‬‬‬‬‬‬‬‬‬‬‬‬‬‬‬‬‬‬‬‬‬‬‬‬‬‬‬‬‬‬‬‬‬‬‬‬‬‬‬‬‬‬‬‬‬‬‬‬‬‬‬‬‬‬‬‬‬‬‬‬‬‬‬‬‬‬‬‬‬‬‬‬‬‬‬‬‬‬‬‬‬‬‬‬‬‬‬‬‬‬‬‬‬‬‬‬‬‬‬‬‬‬‬‬‬‬‬‬‬‬‬‬‬‬‬‬‬‬‬‬‬‬‬‬‬‬‬‬‬‬‬‬‬‬‬‬‬‬‬‬‬‬‬‬‬‬‬‬‬‬‬‬‬‬‬‬‬‬‬‬‬‬‬‬‬‬‬‬‬‬‬‬‬‬‬‬‬‬‬‬‬‬‬‬‬‬‬‬‬‬‬‬‬‬‬‬‬‬‬‬‬‬‬‬‬‬‬‬‬‬‬‬‬‬‬‬‬‬‬‬‬‬‬‬‬‬‬‬‬‬‬‬‬‬‬‬‬‬‬‬‬‬‬‬‬‬‬‬‬‬‬‬‬‬‬‬‬‬‬‬‬‬‬‬‬‬‬‬‬‬‬‬‬‬‬‬‬‬‬‬‬‬‬‬‬‬‬‬‬‬‬‬‬‬‬‬‬‬‬‬‬‬‬‬‬‬‬‬‬‬‬‬‬‬‬‬‬‬‬‬‬‬‬‬‬‬‬‬‬‬‬‬‬‬‬‬‬‬‬‬‬‬‬‬‬‬‬‬‬‬‬‬‬‬‬‬‬‬‬‬‬‬‬‬‬‬‬‬‬‬‬‬‬‬‬‬‬‬‬‬‬‬‬‬‬‬‬‬‬‬‬‬‬‬‬‬‬‬‬‬‬‬‬‬‬‬‬‬‬‬‬‬‬‬‬‬‬‬‬‬‬‬‬‬‬‬‬‬‬‬‬‬‬‬‬‬‬‬‬‬‬‬‬‬‬‬‬‬‬‬‬‬‬‬‬‬‬‬‬‬‬‬‬‬‬‬‬‬‬‬‬‬‬‬‬‬‬‬‬‬‬‬‬‬‬‬‬‬‬‬‬‬‬‬‬‬‬‬‬‬‬‬‬‬‬‬‬‬‬‬‬‬‬‬‬‬‬‬‬‬‬‬‬‬‬‬‬‬‬‬‬‬‬‬‬‬‬‬‬‬‬‬‬‬‬‬‬‬‬‬‬‬‬‬‬‬‬‬‬‬‬‬‬‬‬‬‬‬‬‬‬‬‬‬‬‬‬‬‬‬‬‬‬‬‬‬‬‬‬‬‬‬‬‬‬‬‬‬‬‬‬‬‬‬‬‬‬‬‬‬‬‬‬‬‬‬‬‬‬‬‬‬‬‬‬‬‬‬‬‬‬‬‬‬‬‬‬‬‬‬‬‬‬‬‬‬‬‬‬‬‬‬‬‬‬‬‬‬‬‬‬‬‬‬‬‬‬‬‬‬‬‬‬‬‬‬‬‬‬‬‬‬‬‬‬‬‬‬‬‬‬‬‬‬‬‬‬‬‬‬‬‬‬‬‬‬‬‬‬‬‬‬‬‬‬‬‬‬‬‬‬‬‬‬‬‬‬‬‬‬‬‬‬‬‬‬‬‬‬‬‬‬‬‬‬‬‬‬‬‬‬‬‬‬‬‬‬‬‬‬‬‬‬‬‬‬‬‬‬‬‬‬‬‬‬‬‬‬‬‬‬‬‬‬‬‬‬‬‬‬‬‬‬‬‬‬‬‬‬‬‬‬‬‬‬‬‬‬‬‬‬‬‬‬‬‬‬‬‬‬‬‬‬‬‬‬‬‬‬‬‬‬‬‬‬‬‬‬‬‬‬‬‬‬‬‬‬‬‬‬‬‬‬‬‬‬‬‬‬‬‬‬‬‬‬‬‬‬‬‬‬‬‬‬‬‬‬‬‬‬‬‬‬‬‬‬‬‬‬‬‬‬‬‬‬‬‬‬‬‬‬‬‬‬‬‬‬‬‬‬‬‬‬‬‬‬‬‬‬‬‬‬‬‬‬‬‬‬‬‬‬‬‬‬‬‬‬‬‬‬‬‬‬‬‬‬‬‬‬‬‬‬‬‬‬‬‬‬‬‬‬‬‬‬‬‬‬‬‬‬‬‬‬‬‬‬‬‬‬‬‬‬‬‬‬‬‬‬‬‬‬‬‬‬‬‬‬‬‬‬‬‬‬‬‬‬‬‬‬‬‬‬‬‬‬‬‬‬‬‬‬‬‬‬‬‬‬‬‬‬‬‬‬‬‬‬‬‬‬‬‬‬‬‬‬‬‬‬‬‬‬‬‬‬‬‬‬‬‬‬‬‬‬‬‬‬‬‬‬‬‬‬‬‬‬‬‬‬‬‬‬‬‬‬‬‬‬‬‬‬‬‬‬‬‬‬‬‬‬‬‬‬‬‬‬‬‬‬‬‬‬‬‬‬‬‬‬‬‬‬‬‬‬‬‬‬‬‬‬‬‬‬‬‬‬‬‬‬‬‬‬‬‬‬‬‬‬‬‬‬‬‬‬‬‬‬‬‬‬‬‬‬‬‬‬‬‬‬‬‬‬‬‬‬‬‬‬‬‬‬‬‬‬‬‬‬‬‬‬‬‬‬‬‬‬‬‬‬‬‬‬‬‬‬‬‬‬‬‬‬‬‬‬‬‬‬‬‬‬‬‬‬‬‬‬‬‬‬‬‬‬‬‬‬‬‬‬‬‬‬‬‬‬‬‬‬‬‬‬‬‬‬‬‬‬‬‬‬‬‬‬‬‬‬‬‬‬‬‬‬‬‬‬‬‬‬‬‬‬‬‬‬‬‬‬‬‬‬‬‬‬‬‬‬‬‬‬‬‬‬‬‬‬‬‬‬‬‬‬‬‬‬‬‬‬‬‬‬‬‬‬‬‬‬‬‬‬‬‬‬‬‬‬‬‬‬‬‬‬‬‬‬‬‬‬‬‬‬‬‬‬‬‬‬‬‬‬‬‬‬‬‬‬‬‬‬‬‬‬‬‬‬‬‬‬‬‬‬‬‬‬‬‬‬‬‬‬‬‬‬‬‬‬‬‬‬‬‬‬‬‬‬‬‬‬‬‬‬‬‬‬‬‬‬‬‬‬‬‬‬‬‬‬‬‬‬‬‬‬‬‬‬‬‬‬‬‬‬‬‬‬‬‬‬‬‬‬‬‬‬‬‬‬‬‬‬‬‬‬‬‬‬‬‬‬‬‬‬‬‬‬‬‬‬‬‬‬‬‬‬‬‬‬‬‬‬‬‬‬‬‬‬‬‬‬‬‬‬‬‬‬‬‬‬‬‬‬‬‬‬‬‬‬‬‬‬‬‬‬‬‬‬‬‬‬‬‬‬‬‬‬‬‬‬‬‬‬‬‬‬‬‬‬‬‬‬‬‬‬‬‬‬‬‬‬‬‬‬‬‬‬‬‬‬‬‬‬‬‬‬‬‬‬‬‬‬‬‬‬‬‬‬‬‬‬‬‬‬‬‬‬‬‬‬‬‬‬‬‬‬‬‬‬‬‬‬‬‬‬‬‬‬‬‬‬‬‬‬‬‬‬‬‬‬‬‬‬‬‬‬‬‬‬‬‬‬‬‬‬‬‬‬‬‬‬‬‬‬‬‬‬‬‬‬‬‬‬‬‬‬‬‬‬‬‬‬‬‬‬‬‬‬‬‬‬‬‬‬‬‬‬‬‬‬‬‬‬‬‬‬‬‬‬‬‬‬‬‬‬‬‬‬‬‬‬‬‬‬‬‬‬‬‬‬‬‬‬‬‬‬‬‬‬‬‬‬‬‬‬‬‬‬‬‬‬‬‬‬‬‬‬‬‬‬‬‬‬‬‬‬‬‬‬‬‬‬‬‬‬‬‬‬‬‬‬‬‬‬‬‬‬‬‬‬‬‬‬‬‬‬‬‬‬‬‬‬‬‬‬‬‬‬‬‬‬‬‬‬‬‬‬‬‬‬‬‬‬‬‬‬‬‬‬‬‬‬‬‬‬‬‬‬‬‬‬‬‬‬‬‬‬‬‬‬‬‬‬‬‬‬‬‬‬‬‬‬‬‬‬‬‬‬‬‬‬‬‬‬‬‬‬‬‬‬‬‬‬‬‬‬‬‬‬‬‬‬‬‬‬‬‬‬‬‬‬‬‬‬‬‬‬‬‬‬‬‬‬‬‬‬‬‬‬‬‬‬‬‬‬‬‬‬‬‬‬‬‬‬‬‬‬‬‬‬‬‬‬‬‬‬‬‬‬‬‬‬‬‬‬‬‬‬‬‬‬‬‬‬‬‬‬‬‬‬‬‬‬‬‬‬‬‬‬‬‬‬‬‬‬‬‬‬‬‬‬‬‬‬‬‬‬‬‬‬‬‬‬‬‬‬‬‬‬‬‬‬‬‬‬‬‬‬‬‬‬‬‬‬‬‬‬‬‬‬‬‬‬‬‬‬‬‬‬‬‬‬‬‬‬‬‬‬‬‬‬‬‬‬‬‬‬‬‬‬‬‬‬‬‬‬‬‬‬‬‬‬‬‬‬‬‬‬‬‬‬‬‬‬‬‬‬‬‬‬‬‬‬‬‬‬‬‬‬‬‬‬‬‬‬‬‬‬‬‬‬‬‬‬‬‬‬‬‬‬‬‬‬‬‬‬‬‬‬‬‬‬‬‬‬‬‬‬‬‬‬‬‬‬‬‬‬‬‬‬‬‬‬‬‬‬‬‬‬‬‬‬‬‬‬‬‬‬‬‬‬‬‬‬‬‬‬‬‬‬‬‬‬‬‬‬‬‬‬‬‬‬‬‬‬‬‬‬‬‬‬‬‬‬‬‬‬‬‬‬‬‬‬‬‬‬‬‬‬‬‬‬‬‬‬‬‬‬‬‬‬‬‬‬‬‬‬‬‬‬‬‬‬‬‬‬‬‬‬‬‬‬‬‬‬‬‬‬‬‬‬‬‬‬‬‬‬‬‬‬‬‬‬‬‬‬‬‬‬‬‬‬‬‬‬‬‬‬‬‬‬‬‬‬‬‬‬‬‬‬‬‬‬‬‬‬‬‬‬‬‬‬‬‬‬‬‬‬‬‬‬‬‬‬‬‬‬‬‬‬‬‬‬‬‬‬‬‬‬‬‬‬‬‬‬‬‬‬‬‬‬‬‬‬‬‬‬‬‬‬‬‬‬‬‬‬‬‬‬‬‬‬‬‬‬‬‬‬‬‬‬‬‬‬‬‬‬‬‬‬‬‬‬‬‬‬‬‬‬‬‬‬‬‬‬‬‬‬‬‬‬‬‬‬‬‬‬‬‬‬‬‬‬‬‬‬‬‬‬‬‬‬‬‬‬‬‬‬‬‬‬‬‬‬‬‬‬‬‬‬‬‬‬‬‬‬‬‬‬‬‬‬‬‬‬‬‬‬‬‬‬‬‬‬‬‬‬‬‬‬‬‬‬‬‬‬‬‬‬‬‬‬‬‬‬‬‬‬‬‬‬‬‬‬‬‬‬‬‬‬‬‬‬‬‬‬‬‬‬‬‬‬‬‬‬‬‬‬‬‬‬‬‬‬‬‬‬‬‬‬‬‬‬‬‬‬‬‬‬‬‬‬‬‬‬‬‬‬‬‬‬‬‬‬‬‬‬‬‬‬‬‬‬‬‬‬‬‬‬‬‬‬‬‬‬‬‬‬‬‬‬‬‬‬‬‬‬‬‬‬‬‬‬‬‬‬‬‬‬‬‬‬‬‬‬‬‬‬‬‬‬‬‬‬‬‬‬‬‬‬‬‬‬‬‬‬‬‬‬‬‬‬‬‬‬‬‬‬‬‬‬‬‬‬‬‬‬‬‬‬‬‬‬‬‬‬‬‬‬‬‬‬‬‬‬‬‬‬‬‬‬‬‬‬‬‬‬‬‬‬‬‬‬‬‬‬‬‬‬‬‬‬‬‬‬‬‬‬‬‬‬‬‬‬‬‬‬‬‬‬‬‬‬‬‬‬‬‬‬‬‬‬‬‬‬‬‬‬‬‬‬‬‬‬‬‬‬‬‬‬‬‬‬‬‬‬‬‬‬‬‬‬‬‬‬‬‬‬‬‬‬‬‬‬‬‬‬‬‬‬‬‬‬‬‬‬‬‬‬‬‬‬‬‬‬‬‬‬‬‬‬‬‬‬‬‬‬‬‬‬‬‬‬‬‬‬‬‬‬‬‬‬‬‬‬‬‬‬‬‬‬‬‬‬‬‬‬‬‬‬‬‬‬‬‬‬‬‬‬‬‬‬‬‬‬‬‬‬‬‬‬‬‬‬‬‬‬‬‬‬‬‬‬‬‬‬‬‬‬‬‬‬‬‬‬‬‬‬‬‬‬‬‬‬‬‬‬‬‬‬‬‬‬‬‬‬‬‬‬‬‬‬‬‬‬‬‬‬‬‬‬‬‬‬‬‬‬‬‬‬‬‬‬‬‬‬‬‬‬‬‬‬‬‬‬‬‬‬‬‬‬‬‬‬‬‬‬‬‬‬‬‬‬‬‬‬‬‬‬‬‬‬‬‬‬‬‬‬‬‬‬‬‬‬‬‬‬‬‬‬‬‬‬‬‬‬‬‬‬‬‬‬‬‬‬‬‬‬‬‬‬‬‬‬‬‬‬‬‬‬‬‬‬‬‬‬‬‬‬‬‬‬‬‬‬‬‬‬‬‬‬‬‬‬‬‬‬‬‬‬‬‬‬‬‬‬‬‬‬‬‬‬‬‬‬‬‬‬‬‬‬‬‬‬‬‬‬‬‬‬‬‬‬‬‬‬‬‬‬‬‬‬‬‬‬‬‬‬‬‬‬‬‬‬‬‬‬‬‬‬‬‬‬‬‬‬‬‬‬‬‬‬‬‬‬‬‬‬‬‬‬‬‬‬‬‬‬‬‬‬‬‬‬‬‬‬‬‬‬‬‬‬‬‬‬‬‬‬‬‬‬‬‬‬‬‬‬‬‬‬‬‬‬‬‬‬‬‬‬‬‬‬‬‬‬‬‬‬‬‬‬‬‬‬‬‬‬‬‬‬‬‬‬‬‬‬‬‬‬‬‬‬‬‬‬‬‬‬‬‬‬‬‬‬‬‬‬‬‬‬‬‬‬‬‬‬‬‬‬‬‬‬‬‬‬‬‬‬‬‬‬‬‬‬‬‬‬‬‬‬‬‬‬‬‬‬‬‬‬‬‬‬‬‬‬‬‬‬‬‬‬‬‬‬‬‬‬‬‬‬‬‬‬‬‬‬‬‬‬‬‬‬‬‬‬‬‬‬‬‬‬‬‬‬‬‬‬‬‬‬‬‬‬‬‬‬‬‬‬‬‬‬‬‬‬‬‬‬‬‬‬‬‬‬‬‬‬‬‬‬‬‬‬‬‬‬‬‬‬‬‬‬‬‬‬‬‬‬‬‬‬‬‬‬‬‬‬‬‬‬‬‬‬‬‬‬‬‬‬‬‬‬‬‬‬‬‬‬‬‬‬‬‬‬‬‬‬‬‬‬‬‬‬‬‬‬‬‬‬‬‬‬‬‬‬‬‬‬‬‬‬‬‬‬‬‬‬‬‬‬‬‬‬‬‬‬‬‬‬‬‬‬‬‬‬‬‬‬‬‬‬‬‬‬‬‬‬‬‬‬‬‬‬‬‬‬‬‬‬‬‬‬‬‬‬‬‬‬‬‬‬‬‬‬‬‬‬‬‬‬‬‬‬‬‬‬‬‬‬‬‬‬‬‬‬‬‬‬‬‬‬‬‬‬‬‬‬‬‬‬‬‬‬‬‬‬‬‬‬‬‬‬‬‬‬‬‬‬‬‬‬‬‬‬‬‬‬‬‬‬‬‬‬‬‬‬‬‬‬‬‬‬‬‬‬‬‬‬‬‬‬‬‬‬‬‬‬‬‬‬‬‬‬‬‬‬‬‬‬‬‬‬‬‬‬‬‬‬‬‬‬‬‬‬‬‬‬‬‬‬‬‬‬‬‬‬‬‬‬‬‬‬‬‬‬‬‬‬‬‬‬‬‬‬‬‬‬‬‬‬‬‬‬‬‬‬‬‬‬‬‬‬‬‬‬‬‬‬‬‬‬‬‬‬‬‬‬‬‬‬‬‬‬‬‬‬‬‬‬‬‬‬‬‬‬‬‬‬‬‬‬‬‬‬‬‬‬‬‬‬‬‬‬‬‬‬‬‬‬‬‬‬‬‬‬‬‬‬‬‬‬‬‬‬‬‬‬‬‬‬‬‬‬‬‬‬‬‬‬‬‬‬‬‬‬‬‬‬‬‬‬‬‬‬‬‬‬‬‬‬‬‬‬‬‬‬‬‬‬‬‬‬‬‬‬‬‬‬‬‬‬‬‬‬‬‬‬‬‬‬‬‬‬‬‬‬‬‬‬‬‬‬‬‬‬‬‬‬‬‬‬‬‬‬‬‬‬‬‬‬‬‬‬‬‬‬‬‬‬‬‬‬‬‬‬‬‬‬‬‬‬‬‬‬‬‬‬‬‬‬‬‬‬‬‬‬‬‬‬‬‬‬‬‬‬‬‬‬‬‬‬‬‬‬‬‬‬‬‬‬‬‬‬‬‬‬‬‬‬‬‬‬‬‬‬‬‬‬‬‬‬‬‬‬‬‬‬‬‬‬‬‬‬‬‬‬‬‬‬‬‬‬‬‬‬‬‬‬‬‬‬‬‬‬‬‬‬‬‬‬‬‬‬‬‬‬‬‬‬‬‬‬‬‬‬‬‬‬‬‬‬‬‬‬‬‬‬‬‬‬‬‬‬‬‬‬‬‬‬‬‬‬‬‬‬‬‬‬‬‬‬‬‬‬‬‬‬‬‬‬‬‬‬‬‬‬‬‬‬‬‬‬‬‬‬‬‬‬‬‬‬‬‬‬‬‬‬‬‬‬‬‬‬‬‬‬‬‬‬‬‬‬‬‬‬‬‬‬‬‬‬‬‬‬‬‬‬‬‬‬‬‬‬‬‬‬‬‬‬‬‬‬‬‬‬‬‬‬‬‬‬‬‬‬‬‬‬‬‬‬‬‬‬‬‬‬‬‬‬‬‬‬‬‬‬‬‬‬‬‬‬‬‬‬‬‬‬‬‬‬‬‬‬‬‬‬‬‬‬‬‬‬‬‬‬‬‬‬‬‬‬‬‬‬‬‬‬‬‬‬‬‬‬‬‬‬‬‬‬‬‬‬‬‬‬‬‬‬‬‬‬‬‬‬‬‬‬‬‬‬‬‬‬‬‬‬‬‬‬‬‬‬‬‬‬‬‬‬‬‬‬‬‬‬‬‬‬‬‬‬‬‬‬‬‬‬‬‬‬‬‬‬‬‬‬‬‬‬‬‬‬‬‬‬‬‬‬‬‬‬‬‬‬‬‬‬‬‬‬‬‬‬‬‬‬‬‬‬‬‬‬‬‬‬‬‬‬‬‬‬‬‬‬‬‬‬‬‬‬‬‬‬‬‬‬‬‬‬‬‬‬‬‬‬‬‬‬‬‬‬‬‬‬‬‬‬‬‬‬‬‬‬‬‬‬‬‬‬‬‬‬‬‬‬‬‬‬‬‬‬‬‬‬‬‬‬‬‬‬‬‬‬‬‬‬‬‬‬‬‬‬‬‬‬‬‬‬‬‬‬‬‬‬‬‬‬‬‬‬‬‬‬‬‬‬‬‬‬‬‬‬‬‬‬‬‬‬‬‬‬‬‬‬‬‬‬‬‬‬‬‬‬‬‬‬‬‬‬‬‬‬‬‬‬‬‬‬‬‬‬‬‬‬‬‬‬‬‬‬‬‬‬‬‬‬‬‬‬‬‬‬‬‬‬‬‬‬‬‬‬‬‬‬‬‬‬‬‬‬‬‬‬‬‬‬‬‬‬‬‬‬‬‬‬‬‬‬‬‬‬‬‬‬‬‬‬‬‬‬‬‬‬‬‬‬‬‬‬‬‬‬‬‬‬‬‬‬‬‬‬‬‬‬‬‬‬‬‬‬‬‬‬‬‬‬‬‬‬‬‬‬‬‬‬‬‬‬‬‬‬‬‬‬‬‬‬‬‬‬‬‬‬‬‬‬‬‬‬‬‬‬‬‬‬‬‬‬‬‬‬‬‬‬‬‬‬‬‬‬‬‬‬‬‬‬‬‬‬‬‬‬‬‬‬‬‬‬‬‬‬‬‬‬‬‬‬‬‬‬‬‬‬‬‬‬‬‬‬‬‬‬‬‬‬‬‬‬‬‬‬‬‬‬‬‬‬‬‬‬‬‬‬‬‬‬‬‬‬‬‬‬‬‬‬‬‬‬‬‬‬‬‬‬‬‬‬‬‬‬‬‬‬‬‬‬‬‬‬‬‬‬‬‬‬‬‬‬‬‬‬‬‬‬‬‬‬‬‬‬‬‬‬‬‬‬‬‬‬‬‬‬‬‬‬‬‬‬‬‬‬‬‬‬‬‬‬‬‬‬‬‬‬‬‬‬‬‬‬‬‬‬‬‬‬‬‬‬‬‬‬‬‬‬‬‬‬‬‬‬‬‬‬‬‬‬‬‬‬‬‬‬‬‬‬‬‬‬‬‬‬‬‬‬‬‬‬‬‬‬‬‬‬‬‬‬‬‬‬‬‬‬‬‬‬‬‬‬‬‬‬‬‬‬‬‬‬‬‬‬‬‬‬‬‬‬‬‬‬‬‬‬‬‬‬‬‬‬‬‬‬‬‬‬‬‬‬‬‬‬‬‬‬‬‬‬‬‬‬‬‬‬‬‬‬‬‬‬‬‬‬‬‬‬‬‬‬‬‬‬‬‬‬‬‬‬‬‬‬‬‬‬‬‬‬‬‬‬‬‬‬‬‬‬‬‬‬‬‬‬‬‬‬‬‬‬‬‬‬‬‬‬‬‬‬‬‬‬‬‬‬‬‬‬‬‬‬‬‬‬‬‬‬‬‬‬‬‬‬‬‬‬‬‬‬‬‬‬‬‬‬‬‬‬‬‬‬‬‬‬‬‬‬‬‬‬‬‬‬‬‬‬‬‬‬‬‬‬‬‬‬‬‬‬‬‬‬‬‬‬‬‬‬‬‬‬‬‬‬‬‬‬‬‬‬‬‬‬‬‬‬‬‬‬‬‬‬‬‬‬‬‬‬‬‬‬‬‬‬‬‬‬‬‬‬‬‬‬‬‬‬‬‬‬‬‬‬‬‬‬‬‬‬‬‬‬‬‬‬‬‬‬‬‬‬‬‬‬‬‬‬‬‬‬‬‬‬‬‬‬‬‬‬‬‬‬‬‬‬‬‬‬‬‬‬‬‬‬‬‬‬‬‬‬‬‬‬‬‬‬‬‬‬‬‬‬‬‬‬‬‬‬‬‬‬‬‬‬‬‬‬‬‬‬‬‬‬‬‬‬‬‬‬‬‬‬‬‬‬‬‬‬‬‬‬‬‬‬‬‬‬‬‬‬‬‬‬‬‬‬‬‬‬‬‬‬‬‬‬‬‬‬‬‬‬‬‬‬‬‬‬‬‬‬‬‬‬‬‬‬‬‬‬‬‬‬‬‬‬‬‬‬‬‬‬‬‬‬‬‬‬‬‬‬‬‬‬‬‬‬‬‬‬‬‬‬‬‬‬‬‬‬‬‬‬‬‬‬‬‬‬‬‬‬‬‬‬‬‬‬‬‬‬‬‬‬‬‬‬‬‬‬‬‬‬‬‬‬‬‬‬‬‬‬‬‬‬‬‬‬‬‬‬‬‬‬‬‬‬‬‬‬‬‬‬‬‬‬‬‬‬‬‬‬‬‬‬‬‬‬‬‬‬‬‬‬‬‬‬‬‬‬‬‬‬‬‬‬‬‬‬‬‬‬‬‬‬‬‬‬‬‬‬‬‬‬‬‬‬‬‬‬‬‬‬‬‬‬‬‬‬‬‬‬‬‬‬‬‬‬‬‬‬‬‬‬‬‬‬‬‬‬‬‬‬‬‬‬‬‬‬‬‬‬‬‬‬‬‬‬‬‬‬‬‬‬‬‬‬‬‬‬‬‬‬‬‬‬‬‬‬‬‬‬‬‬‬‬‬‬‬‬‬‬‬‬‬‬‬‬‬‬‬‬‬‬‬‬‬‬‬‬‬‬‬‬‬‬‬‬‬‬‬‬‬‬‬‬‬‬‬‬‬‬‬‬‬‬‬‬‬‬‬‬‬‬‬‬‬‬‬‬‬‬‬‬‬‬‬‬‬‬‬‬‬‬‬‬‬‬‬‬‬‬‬‬‬‬‬‬‬‬‬‬‬‬‬‬‬‬‬‬‬‬‬‬‬‬‬‬‬‬‬‬‬‬‬‬‬‬‬‬‬‬‬‬‬‬‬‬‬‬‬‬‬‬‬‬‬‬‬‬‬‬‬‬‬‬‬‬‬‬‬‬‬‬‬‬‬‬‬‬‬‬‬‬‬‬‬‬‬‬‬‬‬‬‬‬‬‬‬‬‬‬‬‬‬‬‬‬‬‬‬‬‬‬‬‬‬‬‬‬‬‬‬‬‬‬‬‬‬‬‬‬‬‬‬‬‬‬‬‬‬‬‬‬‬‬‬‬‬‬‬‬‬‬‬‬‬‬‬‬‬‬‬‬‬‬‬‬‬‬‬‬‬‬‬‬‬‬‬‬‬‬‬‬‬‬‬‬‬‬‬‬‬‬‬‬‬‬‬‬‬‬‬‬‬‬‬‬‬‬‬‬‬‬‬‬‬‬‬‬‬‬‬‬‬‬‬‬‬‬‬‬‬‬‬‬‬‬‬‬‬‬‬‬‬‬‬‬‬‬‬‬‬‬‬‬‬‬‬‬‬‬‬‬‬‬‬‬‬‬‬‬‬‬‬‬‬‬‬‬‬‬‬‬‬‬‬‬‬‬‬‬‬‬‬‬‬‬‬‬‬‬‬‬‬‬‬‬‬‬‬‬‬‬‬‬‬‬‬‬‬‬‬‬‬‬‬‬‬‬‬‬‬‬‬‬‬‬‬‬‬‬‬‬‬‬‬‬‬‬‬‬‬‬‬‬‬‬‬‬‬‬‬‬‬‬‬‬‬‬‬‬‬‬‬‬‬‬‬‬‬‬‬‬‬‬‬‬‬‬‬‬‬‬‬‬‬‬‬‬‬‬‬‬‬‬‬‬‬‬‬‬‬‬‬‬‬‬‬‬‬‬‬‬‬‬‬‬‬‬‬‬‬‬‬‬‬‬‬‬‬‬‬‬‬‬‬‬‬‬‬‬‬‬‬‬‬‬‬‬‬‬‬‬‬‬‬‬‬‬‬‬‬‬‬‬‬‬‬‬‬‬‬‬‬‬‬‬‬‬‬‬‬‬‬‬‬‬‬‬‬‬‬‬‬‬‬‬‬‬‬‬‬‬‬‬‬‬‬‬‬‬‬‬‬‬‬‬‬‬‬‬‬‬‬‬‬‬‬‬‬‬‬‬‬‬‬‬‬‬‬‬‬‬‬‬‬‬‬‬‬‬‬‬‬‬‬‬‬‬‬‬‬‬‬‬‬‬‬‬‬‬‬‬‬‬‬‬‬‬‬‬‬‬‬‬‬‬‬‬‬‬‬‬‬‬‬‬‬‬‬‬‬‬‬‬‬‬‬‬‬‬‬‬‬‬‬‬‬‬‬‬‬‬‬‬‬‬‬‬‬‬‬‬‬‬‬‬‬‬‬‬‬‬‬‬‬‬‬‬‬‬‬‬‬‬‬‬‬‬‬‬‬‬‬‬‬‬‬‬‬‬‬‬‬‬‬‬‬‬‬‬‬‬‬‬‬‬‬‬‬‬‬‬‬‬‬‬‬‬‬‬‬‬‬‬‬‬‬‬‬‬‬‬‬‬‬‬‬‬‬‬‬‬‬‬‬‬‬‬‬‬‬‬‬‬‬‬‬‬‬‬‬‬‬‬‬‬‬‬‬‬‬‬‬‬‬‬‬‬‬‬‬‬‬‬‬‬‬‬‬‬‬‬‬‬‬‬‬‬‬‬‬‬‬‬‬‬‬‬‬‬‬‬‬‬‬‬‬‬‬‬‬‬‬‬‬‬‬‬‬‬‬‬‬‬‬‬‬‬‬‬‬‬‬‬‬‬‬‬‬‬‬‬‬‬‬‬‬‬‬‬‬‬‬‬‬‬‬‬‬‬‬‬‬‬‬‬‬‬‬‬‬‬‬‬‬‬‬‬‬‬‬‬‬‬‬‬‬‬‬‬‬‬‬‬‬‬‬‬‬‬‬‬‬‬‬‬‬‬‬‬‬‬‬‬‬‬‬‬‬‬‬‬‬‬‬‬‬‬‬‬‬‬‬‬‬‬‬‬‬‬‬‬‬‬‬‬‬‬‬‬‬‬‬‬‬‬‬‬‬‬‬‬‬‬‬‬‬‬‬‬‬‬‬‬‬‬‬‬‬‬‬‬‬‬‬‬‬‬‬‬‬‬‬‬‬‬‬‬‬‬‬‬‬‬‬‬‬‬‬‬‬‬‬‬‬‬‬‬‬‬‬‬‬‬‬‬‬‬‬‬‬‬‬‬‬‬‬‬‬‬‬‬‬‬‬‬‬‬‬‬‬‬‬‬‬‬‬‬‬‬‬‬‬‬‬‬‬‬‬‬‬‬‬‬‬‬‬‬‬‬‬‬‬‬‬‬‬‬‬‬‬‬‬‬‬‬‬‬‬‬‬‬‬‬‬‬‬‬‬‬‬‬‬‬‬‬‬‬‬‬‬‬‬‬‬‬‬‬‬‬‬‬‬‬‬‬‬‬‬‬‬‬‬‬‬‬‬‬‬‬‬‬‬‬‬‬‬‬‬‬‬‬‬‬‬‬‬‬‬‬‬‬‬‬‬‬‬‬‬‬‬‬‬‬‬‬‬‬‬‬‬‬‬‬‬‬‬‬‬‬‬‬‬‬‬‬‬‬‬‬‬‬‬‬‬‬‬‬‬‬‬‬‬‬‬‬‬‬‬‬‬‬‬‬‬‬‬‬‬‬‬‬‬‬‬‬‬‬‬‬‬‬‬‬‬‬‬‬‬‬‬‬‬‬‬‬‬‬‬‬‬‬‬‬‬‬‬‬‬‬‬‬‬‬‬‬‬‬‬‬‬‬‬‬‬‬‬‬‬‬‬‬‬‬‬‬‬‬‬‬‬‬‬‬‬‬‬‬‬‬‬‬‬‬‬‬‬‬‬‬‬‬‬‬‬‬‬‬‬‬‬‬‬‬‬‬‬‬‬‬‬‬‬‬‬‬‬‬‬‬‬‬‬‬‬‬‬‬‬‬‬‬‬‬‬‬‬‬‬‬‬‬‬‬‬‬‬‬‬‬‬‬‬‬‬‬‬‬‬‬‬‬‬‬‬‬‬‬‬‬‬‬‬‬‬‬‬‬‬‬‬‬‬‬‬‬‬‬‬‬‬‬‬‬‬‬‬‬‬‬‬‬‬‬‬‬‬‬‬‬‬‬‬‬‬‬‬‬‬‬‬‬‬‬‬‬‬‬‬‬‬‬‬‬‬‬‬‬‬‬‬‬‬‬‬‬‬‬‬‬‬‬‬‬‬‬‬‬‬‬‬‬‬‬‬‬‬‬‬‬‬‬‬‬‬‬‬‬‬‬‬‬‬‬‬‬‬‬‬‬‬‬‬‬‬‬‬‬‬‬‬‬‬‬‬‬‬‬‬‬‬‬‬‬‬‬‬‬‬‬‬‬‬‬‬‬‬‬‬‬‬‬‬‬‬‬‬‬‬‬‬‬‬‬‬‬‬‬‬‬‬‬‬‬‬‬‬‬‬‬‬‬‬‬‬‬‬‬‬‬‬‬‬‬‬‬‬‬‬‬‬‬‬‬‬‬‬‬‬‬‬‬‬‬‬‬‬‬‬‬‬‬‬‬‬‬‬‬‬‬‬‬‬‬‬‬‬‬‬‬‬‬‬‬‬‬‬‬‬‬‬‬‬‬‬‬‬‬‬‬‬‬‬‬‬‬‬‬‬‬‬‬‬‬‬‬‬‬‬‬‬‬‬‬‬‬‬‬‬‬‬‬‬‬‬‬‬‬‬‬‬‬‬‬‬‬‬‬‬‬‬‬‬‬‬‬‬‬‬‬‬‬‬‬‬‬‬‬‬‬‬‬‬‬‬‬‬‬‬‬‬‬‬‬‬‬‬‬‬‬‬‬‬‬‬‬‬‬‬‬‬‬‬‬‬‬‬‬‬‬‬‬‬‬‬‬‬‬‬‬‬‬‬‬‬‬‬‬‬‬‬‬‬‬‬‬‬‬‬‬‬‬‬‬‬‬‬‬‬‬‬‬‬‬‬‬‬‬‬‬‬‬‬‬‬‬‬‬‬‬‬‬‬‬‬‬‬‬‬‬‬‬‬‬‬‬‬‬‬‬‬‬‬‬‬‬‬‬‬‬‬‬‬‬‬‬‬‬‬‬‬‬‬‬‬‬‬‬‬‬‬‬‬‬‬‬‬‬‬‬‬‬‬‬‬‬‬‬‬‬‬‬‬‬‬‬‬‬‬‬‬‬‬‬‬‬‬‬‬‬‬‬‬‬‬‬‬‬‬‬‬‬‬‬‬‬‬‬‬‬‬‬‬‬‬‬‬‬‬‬‬‬‬‬‬‬‬‬‬‬‬‬‬‬‬‬‬‬‬‬‬‬‬‬‬‬‬‬‬‬‬‬‬‬‬‬‬‬‬‬‬‬‬‬‬‬‬‬‬‬‬‬‬‬‬‬‬‬‬‬‬‬‬‬‬‬‬‬‬‬‬‬‬‬‬‬‬‬‬‬‬‬‬‬‬‬‬‬‬‬‬‬‬‬‬‬‬‬‬‬‬‬‬‬‬‬‬‬‬‬‬‬‬‬‬‬‬‬‬‬‬‬‬‬‬‬‬‬‬‬‬‬‬‬‬‬‬‬‬‬‬‬‬‬‬‬‬‬‬‬‬‬‬‬‬‬‬‬‬‬‬‬‬‬‬‬‬‬‬‬‬‬‬‬‬‬‬‬‬‬‬‬‬‬‬‬‬‬‬‬‬‬‬‬‬‬‬‬‬‬‬‬‬‬‬‬‬‬‬‬‬‬‬‬‬‬‬‬‬‬‬‬‬‬‬‬‬‬‬‬‬‬‬‬‬‬‬‬‬‬‬‬‬‬‬‬‬‬‬‬‬‬‬‬‬‬‬‬‬‬‬‬‬‬‬‬‬‬‬‬‬‬‬‬‬‬‬‬‬‬‬‬‬‬‬‬‬‬‬‬‬‬‬‬‬‬‬‬‬‬‬‬‬‬‬‬‬‬‬‬‬‬‬‬‬‬‬‬‬‬‬‬‬‬‬‬‬‬‬‬‬‬‬‬‬‬‬‬‬‬‬‬‬‬‬‬‬‬‬‬‬‬‬‬‬‬‬‬‬‬‬‬‬‬‬‬‬‬‬‬‬‬‬‬‬‬‬‬‬‬‬‬‬‬‬‬‬‬‬‬‬‬‬‬‬‬‬‬‬‬‬‬‬‬‬‬‬‬‬‬‬‬‬‬‬‬‬‬‬‬‬‬‬‬‬‬‬‬‬‬‬‬‬‬‬‬‬‬‬‬‬‬‬‬‬‬‬‬‬‬‬‬‬‬‬‬‬‬‬‬‬‬‬‬‬‬‬‬‬‬‬‬‬‬‬‬‬‬‬‬‬‬‬‬‬‬‬‬‬‬‬‬‬‬‬‬‬‬‬‬‬‬‬‬‬‬‬‬‬‬‬‬‬‬‬‬‬‬‬‬‬‬‬‬‬‬‬‬‬‬‬‬‬‬‬‬‬‬‬‬‬‬‬‬‬‬‬‬‬‬‬‬‬‬‬‬‬‬‬‬‬‬‬‬‬‬‬‬‬‬‬‬‬‬‬‬‬‬‬‬‬‬‬‬‬‬‬‬‬‬‬‬‬‬‬‬‬‬‬‬‬‬‬‬‬‬‬‬‬‬‬‬‬‬‬‬‬‬‬‬‬‬‬‬‬‬‬‬‬‬‬‬‬‬‬‬‬‬‬‬‬‬‬‬‬‬‬‬‬‬‬‬‬‬‬‬‬‬‬‬‬‬‬‬‬‬‬‬‬‬‬‬‬‬‬‬‬‬‬‬‬‬‬‬‬‬‬‬‬‬‬‬‬‬‬‬‬‬‬‬‬‬‬‬‬‬‬‬‬‬‬‬‬‬‬‬‬‬‬‬‬‬‬‬‬‬‬‬‬‬‬‬‬‬‬‬‬‬‬‬‬‬‬‬‬‬‬‬‬‬‬‬‬‬‬‬‬‬‬‬‬‬‬‬‬‬‬‬‬‬‬‬‬‬‬‬‬‬‬‬‬‬‬‬‬‬‬‬‬‬‬‬‬‬‬‬‬‬‬‬‬‬‬‬‬‬‬‬‬‬‬‬‬‬‬‬‬‬‬‬‬‬‬‬‬‬‬‬‬‬‬‬‬‬‬‬‬‬‬‬‬‬‬‬‬‬‬‬‬‬‬‬‬‬‬‬‬‬‬‬‬‬‬‬‬‬‬‬‬‬‬‬‬‬‬‬‬‬‬‬‬‬‬‬‬‬‬‬‬‬‬‬‬‬‬‬‬‬‬‬‬‬‬‬‬‬‬‬‬‬‬‬‬‬‬‬‬‬‬‬‬‬‬‬‬‬‬‬‬‬‬‬‬‬‬‬‬‬‬‬‬‬‬‬‬‬‬‬‬‬‬‬‬‬‬‬‬‬‬‬‬‬‬‬‬‬‬‬‬‬‬‬‬‬‬‬‬‬‬‬‬‬‬‬‬‬‬‬‬‬‬‬‬‬‬‬‬‬‬‬‬‬‬‬‬‬‬‬‬‬‬‬‬‬‬‬‬‬‬‬‬‬‬‬‬‬‬‬‬‬‬‬‬‬‬‬‬‬‬‬‬‬‬‬‬‬‬‬‬‬‬‬‬‬‬‬‬‬‬‬‬‬‬‬‬‬‬‬‬‬‬‬‬‬‬‬‬‬‬‬‬‬‬‬‬‬‬‬‬‬‬‬‬‬‬‬‬‬‬‬‬‬‬‬‬‬‬‬‬‬‬‬‬‬‬‬‬‬‬‬‬‬‬‬‬‬‬‬‬‬‬‬‬‬‬‬‬‬‬‬‬‬‬‬‬‬‬‬‬‬‬‬‬‬‬‬‬‬‬‬‬‬‬‬‬‬‬‬‬‬‬‬‬‬‬‬‬‬‬‬‬‬‬‬‬‬‬‬‬‬‬‬‬‬‬‬‬‬‬‬‬‬‬‬‬‬‬‬‬‬‬‬‬‬‬‬‬‬‬‬‬‬‬‬‬‬‬‬‬‬‬‬‬‬‬‬‬‬‬‬‬‬‬‬‬‬‬‬‬‬‬‬‬‬‬‬‬‬‬‬‬‬‬‬‬‬‬‬‬‬‬‬‬‬‬‬‬‬‬‬‬‬‬‬‬‬‬‬‬‬‬‬‬‬‬‬‬‬‬‬‬‬‬‬‬‬‬‬‬‬‬‬‬‬‬‬‬‬‬‬‬‬‬‬‬‬‬‬‬‬‬‬‬‬‬‬‬‬‬‬‬‬‬‬‬‬‬‬‬‬‬‬‬‬‬‬‬‬‬‬‬‬‬‬‬‬‬‬‬‬‬‬‬‬‬‬‬‬‬‬‬‬‬‬‬‬‬‬‬‬‬‬‬‬‬‬‬‬‬‬‬‬‬‬‬‬‬‬‬‬‬‬‬‬‬‬‬‬‬‬‬‬‬‬‬‬‬‬‬‬‬‬‬‬‬‬‬‬‬‬‬‬‬‬‬‬‬‬‬‬‬‬‬‬‬‬‬‬‬‬‬‬‬‬‬‬‬‬‬‬‬‬‬‬‬‬‬‬‬‬‬‬‬‬‬‬‬‬‬‬‬‬‬‬‬‬‬‬‬‬‬‬‬‬‬‬‬‬‬‬‬‬‬‬‬‬‬‬‬‬‬‬‬‬‬‬‬‬‬‬‬‬‬‬‬‬‬‬‬‬‬‬‬‬‬‬‬‬‬‬‬‬‬‬‬‬‬‬‬‬‬‬‬‬‬‬‬‬‬‬‬‬‬‬‬‬‬‬‬‬‬‬‬‬‬‬‬‬‬‬‬‬‬‬‬‬‬‬‬‬‬‬‬‬‬‬‬‬‬‬‬‬‬‬‬‬‬‬‬‬‬‬‬‬‬‬‬‬‬‬‬‬‬‬‬‬‬‬‬‬‬‬‬‬‬‬‬‬‬‬‬‬‬‬‬‬‬‬‬‬‬‬‬‬‬‬‬‬‬‬‬‬‬‬‬‬‬‬‬‬‬‬‬‬‬‬‬‬‬‬‬‬‬‬‬‬‬‬‬‬‬‬‬‬‬‬‬‬‬‬‬‬‬‬‬‬‬‬‬‬‬‬‬‬‬‬‬‬‬‬‬‬‬‬‬‬‬‬‬‬‬‬‬‬‬‬‬‬‬‬‬‬‬‬‬‬‬‬‬‬‬‬‬‬‬‬‬‬‬‬‬‬‬‬‬‬‬‬‬‬‬‬‬‬‬‬‬‬‬‬‬‬‬‬‬‬‬‬‬‬‬‬‬‬‬‬‬‬‬‬‬‬‬‬‬‬‬‬‬‬‬‬‬‬‬‬‬‬‬‬‬‬‬‬‬‬‬‬‬‬‬‬‬‬‬‬‬‬‬‬‬‬‬‬‬‬‬‬‬‬‬‬‬‬‬‬‬‬‬‬‬‬‬‬‬‬‬‬‬‬‬‬‬‬‬‬‬‬‬‬‬‬‬‬‬‬‬‬‬‬‬‬‬‬‬‬‬‬‬‬‬‬‬‬‬‬‬‬‬‬‬‬‬‬‬‬‬‬‬‬‬‬‬‬‬‬‬‬‬‬‬‬‬‬‬‬‬‬‬‬‬‬‬‬‬‬‬‬‬‬‬‬‬‬‬‬‬‬‬‬‬‬‬‬‬‬‬‬‬‬‬‬‬‬‬‬‬‬‬‬‬‬‬‬‬‬‬‬‬‬‬‬‬‬‬‬‬‬‬‬‬‬‬‬‬‬‬‬‬‬‬‬‬‬‬‬‬‬‬‬‬‬‬‬‬‬‬‬‬‬‬‬‬‬‬‬‬‬‬‬‬‬‬‬‬‬‬‬‬‬‬‬‬‬‬‬‬‬‬‬‬‬‬‬‬‬‬‬‬‬‬‬‬‬‬‬‬‬‬‬‬‬‬‬‬‬‬‬‬‬‬‬‬‬‬‬‬‬‬‬‬‬‬‬‬‬‬‬‬‬‬‬‬‬‬‬‬‬‬‬‬‬‬‬‬‬‬‬‬‬‬‬‬‬‬‬‬‬‬‬‬‬‬‬‬‬‬‬‬‬‬‬‬‬‬‬‬‬‬‬‬‬‬‬‬‬‬‬‬‬‬‬‬‬‬‬‬‬‬‬‬‬‬‬‬‬‬‬‬‬‬‬‬‬‬‬‬‬‬‬‬‬‬‬‬‬‬‬‬‬‬‬‬‬‬‬‬‬‬‬‬‬‬‬‬‬‬‬‬‬‬‬‬‬‬‬‬‬‬‬‬‬‬‬‬‬‬‬‬‬‬‬‬‬‬‬‬‬‬‬‬‬‬‬‬‬‬‬‬‬‬‬‬‬‬‬‬‬‬‬‬‬‬‬‬‬‬‬‬‬‬‬‬‬‬‬‬‬‬‬‬‬‬‬‬‬‬‬‬‬‬‬‬‬‬‬‬‬‬‬‬‬‬‬‬‬‬‬‬‬‬‬‬‬‬‬‬‬‬‬‬‬‬‬‬‬‬‬‬‬‬‬‬‬‬‬‬‬‬‬‬‬‬‬‬‬‬‬‬‬‬‬‬‬‬‬‬‬‬‬‬‬‬‬‬‬‬‬‬‬‬‬‬‬‬‬‬‬‬‬‬‬‬‬‬‬‬‬‬‬‬‬‬‬‬‬‬‬‬‬‬‬‬‬‬‬‬‬‬‬‬‬‬‬‬‬‬‬‬‬‬‬‬‬‬‬‬‬‬‬‬‬‬‬‬‬‬‬‬‬‬‬‬‬‬‬‬‬‬‬‬‬‬‬‬‬‬‬‬‬‬‬‬‬‬‬‬‬‬‬‬‬‬‬‬‬‬‬‬‬‬‬‬‬‬‬‬‬‬‬‬‬‬‬‬‬‬‬‬‬‬‬‬‬‬‬‬‬‬‬‬‬‬‬‬‬‬‬‬‬‬‬‬‬‬‬‬‬‬‬‬‬‬‬‬‬‬‬‬‬‬‬‬‬‬‬‬‬‬‬‬‬‬‬‬‬‬‬‬‬‬‬‬‬‬‬‬‬‬‬‬‬‬‬‬‬‬‬‬‬‬‬‬‬‬‬‬‬‬‬‬‬‬‬‬‬‬‬‬‬‬‬‬‬‬‬‬‬‬‬‬‬‬‬‬‬‬‬‬‬‬‬‬‬‬‬‬‬‬‬‬‬‬‬‬‬‬‬‬‬‬‬‬‬‬‬‬‬‬‬‬‬‬‬‬‬‬‬‬‬‬‬‬‬‬‬‬‬‬‬‬‬‬‬‬‬‬‬‬‬‬‬‬‬‬‬‬‬‬‬‬‬‬‬‬‬‬‬‬‬‬‬‬‬‬‬‬‬‬‬‬‬‬‬‬‬‬‬‬‬‬‬‬‬‬‬‬‬‬‬‬‬‬‬‬‬‬‬‬‬‬‬‬‬‬‬‬‬‬‬‬‬‬‬‬‬‬‬‬‬‬‬‬‬‬‬‬‬‬‬‬‬‬‬‬‬‬‬‬‬‬‬‬‬‬‬‬‬‬‬‬‬‬‬‬‬‬‬‬‬‬‬‬‬‬‬‬‬‬‬‬‬‬‬‬‬‬‬‬‬‬‬‬‬‬‬‬‬‬‬‬‬‬‬‬‬‬‬‬‬‬‬‬‬‬‬‬‬‬‬‬‬‬‬‬‬‬‬‬‬‬‬‬‬‬‬‬‬‬‬‬‬‬‬‬‬‬‬‬‬‬‬‬‬‬‬‬‬‬‬‬‬‬‬‬‬‬‬‬‬‬‬‬‬‬‬‬‬‬‬‬‬‬‬‬‬‬‬‬‬‬‬‬‬‬‬‬‬‬‬‬‬‬‬‬‬‬‬‬‬‬‬‬‬‬‬‬‬‬‬‬‬‬‬‬‬‬‬‬‬‬‬‬‬‬‬‬‬‬‬‬‬‬‬‬‬‬‬‬‬‬‬‬‬‬‬‬‬‬‬‬‬‬‬‬‬‬‬‬‬‬‬‬‬‬‬‬‬‬‬‬‬‬‬‬‬‬‬‬‬‬‬‬‬‬‬‬‬‬‬‬‬‬‬‬‬‬‬‬‬‬‬‬‬‬‬‬‬‬‬‬‬‬‬‬‬‬‬‬‬‬‬‬‬‬‬‬‬‬‬‬‬‬‬‬‬‬‬‬‬‬‬‬‬‬‬‬‬‬‬‬‬‬‬‬‬‬‬‬‬‬‬‬‬‬‬‬‬‬‬‬‬‬‬‬‬‬‬‬‬‬‬‬‬‬‬‬‬‬‬‬‬‬‬‬‬‬‬‬‬‬‬‬‬‬‬‬‬‬‬‬‬‬‬‬‬‬‬‬‬‬‬‬‬‬‬‬‬‬‬‬‬‬‬‬‬‬‬‬‬‬‬‬‬‬‬‬‬‬‬‬‬‬‬‬‬‬‬‬‬‬‬‬‬‬‬‬‬‬‬‬‬‬‬‬‬‬‬‬‬‬‬‬‬‬‬‬‬‬‬‬‬‬‬‬‬‬‬‬‬‬‬‬‬‬‬‬‬‬‬‬‬‬‬‬‬‬‬‬‬‬‬‬‬‬‬‬‬‬‬‬‬‬‬‬‬‬‬‬‬‬‬‬‬‬‬‬‬‬‬‬‬‬‬‬‬‬‬‬‬‬‬‬‬‬‬‬‬‬‬‬‬‬‬‬‬‬‬‬‬‬‬‬‬‬‬‬‬‬‬‬‬‬‬‬‬‬‬‬‬‬‬‬‬‬‬‬‬‬‬‬‬‬‬‬‬‬‬‬‬‬‬‬‬‬‬‬‬‬‬‬‬‬‬‬‬‬‬‬‬‬‬‬‬‬‬‬‬‬‬‬‬‬‬‬‬‬‬‬‬‬‬‬‬‬‬‬‬‬‬‬‬‬‬‬‬‬‬‬‬‬‬‬‬‬‬‬‬‬‬‬‬‬‬‬‬‬‬‬‬‬‬‬‬‬‬‬‬‬‬‬‬‬‬‬‬‬‬‬‬‬‬‬‬‬‬‬‬‬‬‬‬‬‬‬‬‬‬‬‬‬‬‬‬‬‬‬‬‬‬‬‬‬‬‬‬‬‬‬‬‬‬‬‬‬‬‬‬‬‬‬‬‬‬‬‬‬‬‬‬‬‬‬‬‬‬‬‬‬‬‬‬‬‬‬‬‬‬‬‬‬‬‬‬‬‬‬‬‬‬‬‬‬‬‬‬‬‬‬‬‬‬‬‬‬‬‬‬‬‬‬‬‬‬‬‬‬‬‬‬‬‬‬‬‬‬‬‬‬‬‬‬‬‬‬‬‬‬‬‬‬‬‬‬‬‬‬‬‬‬‬‬‬‬‬‬‬‬‬‬‬‬‬‬‬‬‬‬‬‬‬‬‬‬‬‬‬‬‬‬‬‬‬‬‬‬‬‬‬‬‬‬‬‬‬‬‬‬‬‬‬‬‬‬‬‬‬‬‬‬‬‬‬‬‬‬‬‬‬‬‬‬‬‬‬‬‬‬‬‬‬‬‬‬‬‬‬‬‬‬‬‬‬‬‬‬‬‬‬‬‬

This study also assessed the concentration of heavy metals (HMs) in sediment samples at different depths. We found that the average concentration of HMs in mangrove sediment samples ranked from the highest to the lowest were iron (Fe; 9.49 ppm) > cobalt (Co; 2.44 ppm) > lead (Pb; 1.86 ppm) > manganese (Mn; 1.37 ppm) > cadmium (Cd; 0.62 ppm) > zinc (Zn; 0.61 ppm) > copper (Cu; 0.11 ppm). This ranking corresponds to that of Nimnoi and Pongsilp (2022) and Lertprasert (2006), who found that the concentration of HMs in mangrove soils along the upper Gulf of Thailand and sediment of the Phi Lok Canal system of Samutsongkhram province, Thailand, were ranked from highest to lowest: Fe > Zn > Cu. The HMs in mangrove ecosystems are mainly from human activities such as chemical dumping, shipping, industrial waste, and urban runoff (Macfarlane and Burchett 2001; Caregnato et al. 2008). The observed concentrations of HMs in sediment samples were below the permissible limits in agricultural soils as per the standards of the World Health Organization (WHO) (Ogunlana et al. 2020). This means that the mangrove forest sites of Hamata, Mangrove Bay, and Saffaga are still free from contamination for the aforementioned HMs, and caution must be taken to avoid potential contamination. This is because the accumulation of HMs in the soil or aquatic environments through surface runoff increases the risk of their absorption by living organisms, leading to cell toxicity and neurodegenerative diseases (Rehman et al. 2021; Ohiagu et al. 2022; Jomova et al. 2025). Overall, our results on the HM concentration in mangrove sediment samples are somewhat comparable to those of El-said & Youssef (2013) and El-Sorogy et al. (2024) who carried out similar studies along the Egyptian Red Sea coast.

In this study, we also found the relation of HMs to bacterial diversity in each sediment sample collected from the studied sampling sites. Some HMs have been found to play a significant role in bacterial metabolic pathways, whereas others are toxic or non-essential (Igiri et al. 2018; Henao and Ghneim-herrera 2021; Orji et al. 2021). Moreover, bacteria have been observed to convert toxic metal ions into their respective non-toxic forms via an ATP-dependent pathway (Igiri et al. 2018; Thai et al. 2023; Fatima et al. 2024; Tang et al. 2024). *Actinomycetes* were found to be influenced by high concentrations of Co in Saffaga (Fig. 5a). This group of microbes is not only known for its production of antibiotics and other useful metabolites but also expresses plasmid-encoded genes involved in heavy metal resistance via several strategies, such as enzymatic detoxification, active transport efflux pumps, exclusion by permeability barriers, as well as intra- and extracellular sequestration (Lin et al. 2021; Gillieatt and Coleman 2024; Nnaji et al. 2024). *Negativicutes* belonging to the phylum *Bacillota* were found to be influenced by Cd, which is one of the global and most serious HM contaminants. *Bacillota* have been reported to induce Cd resistance or participate in Cd immobilization in contaminated soils (Li et al. 2021b). The application of *Bacillota* could aid in the bioremediation of Cd-contaminated soils (Ma et al. 2023). *Prevotella,* which belongs to the phylum *Bacteroidota* were found to be influenced by Pb. *Bacteroidota* is also one of the most dominant bacterial phyla in heavy metal-contaminated environments. This is because most of the species in *Bacteroidota* can generate nutrients from their chemoheterotrophic pathways and, thus, are relatively tolerant to HM stress (Li et al. 2022a).

The metagenomics dataset of sediment samples in this study revealed that the numbers of observed species and bacterial diversity and richness in sampling sites of Hamata, Mangrove Bay, and Saffaga were not significantly different from each other, mainly due to the similarity in dominant mangrove species and mixed land use. All sites were utilized in different categories of land use, such as urbanization, agriculture, and tourism. *Pseudomonadota* (synonym “*Pseudomonadota*”) was the dominant phylum, followed by *Bacteroidota* (synonym “*Bacteroidota*”), *Bacillota_A_368345* and *Bacillota_I* (synonym “*Bacillota*”), *Actinomycetota* (synonym “*Actinobacteria*”), and *Chloroflexota* (synonym “*Chloroflexi*”). This result corresponds to previous studies on mangrove ecosystems. For instance, Nimnoi and Pongslip (2022) reported that the phylum *Pseudomonadota* was the most abundant, followed by *Desulfobacterota*, *Bacteroidota*, *Chloroflexi*, *Crenarchaeota*, *Acidobacteriota*, *Bacillota*, *Myxococcota*, *Gemmatimonadota*, and *Halobacterota* in mangrove forest soils along the upper Gulf of Thailand. Alghamdi et al. (2024) observed *Pseudomonadota*, *Bacillota*, *Desulfobacterota*, *Bacteroidota*, *Actinobacteriota*, *Myxococcota*, and *Gemmatimonadota* as the most dominant phyla in *A. marina* soil that was collected along the Saudi Arabian Red Sea coast. In another study, Thomson et al. (2022) investigated the influence of seasonal variation on the diversity and composition of bacteria in *A. marina* soils and reported *Pseudomonadota*, *Chloroflexi*, and *Bacteroidota* as the most dominant phyla in the surface and subsurface soils in both summer and winter seasons. In *Rhizophora mucronata* soils, Allaouia et al. (2022) found *Pseudomonadota*, *Desulfobacteria*, *Bacteroidota*, and *Chloroflexota* as some of the most abundant phyla. In the Indian mangrove system, Ghosh et al. (2022) reported *Pseudomonadota*, *Bacillota*, *Bacteroidota*, and *Actinobacteria* as the most abundant phyla. Overall, these results indicate that *Pseudomonadota* is the most predominant and cosmopolitan bacterial phylum in diverse mangrove environments. *Pseudomonadota* are metabolically diverse and can survive in different ecosystems due to the presence of different groups of genes in their DNA responsible for stress resistance (Ghosh et al. 2022). On the other hand, we also observed a significant relative abundance of unclassified bacteria at the phylum and class levels in HR and MA sediment samples. Unclassified bacteria are common in environmental microbiome analysis (Pu et al. 2024). Delgado-Baquerizo (2019) conducted a global soil survey of 235 sites and concluded that 99% of bacterial phylotypes could not be taxonomically classified, especially in boreal and tropical forests. The authors attributed this to environmental factors such as precipitation, temperature, pH, and soil nutrient content and texture that could lead to an increased abundance of these unclassified microbes. Soil nutrient and texture analysis revealed high concentrations of nitrogen in HR and MA sediment samples (Supplementary Table 1), which could have led to this observation. High nitrogen levels influence microbial composition in sediments, potentially leading to a higher abundance of unclassified bacteria. This is because excess nitrogen alters the sediment’s chemical properties, which in turn favors the proliferation of certain bacterial groups while potentially hindering others. These shifts in microbial community structure can be observed through changes in the relative abundance of different bacterial phyla (Wang et al. 2018; Lin et al. 2019).

Our analysis revealed significant variations in microbial community composition across the study sites (Fig. 3a, b). For instance, the high abundance of *Pseudomonadota* (67.6%) at Mangrove Bay (MA) and *Bacteroidota* (45.9%) at Saffaga (SA) suggests that local site conditions are shaping distinct communities. These differences correlate with variations in environmental factors. The redundancy analysis (RDA) (Fig. 5) showed that the high levels of copper at Mangrove Bay strongly influenced its microbial structure, which was dominated by copper-tolerant genera like *Cognatiicoccus* and *Colwellia_A* (Supplementary Table 2). Similarly, the Saffaga sites (SA, SR) showed elevated cobalt levels, which correlated with a higher abundance of *Actinomycetota* (Fig. 5a), a phylum known to include metal-resistant members. These shifts in community structure are directly reflected in the metabolic potential for each community. The high abundance of *Pseudomonadota* and *Bacteroidota* across all sites, which are known to be metabolically versatile, explains the dominance of broad metabolic functions like “aerobic chemoheterotrophy” and “fermentation” observed in the FAPROTAX analysis (Fig. 6a).

Gammaproteobacteria and Alphaproteobacteria were the most dominant classes in SR and MA sediment samples, respectively (Fig. 3b), and these have been reported to play a vital role in biogeochemical cycles, especially in the nitrogen and carbon cycle (Dyksma et al. 2016; Goethem et al. 2017). The class *Bacteroidia*, which was more dominant in SA, has been previously reported for its attributes, such as wide distribution in different environments (sediments, soil, and seawater), the ability to biosynthesize acetic and succinic acid and proteolytic activity (Mendes et al. 2013; Nimnoi and Pongsilp 2022). Microbial biosynthesis of succinic acid via the reverse TCA (Tricarboxylic acid cycle) promotes carbon dioxide fixation, thus an important process in carbon sequestration (Malubhoy et al. 2022). *Clostridia,* which are the dominant class in HA, include bacterial genera that have been previously reported to play a role in the sulfur cycle (Sallam and Steinbu 2009). The predicted functional correlation with HMs showed high positive correlations of Co to fermentation and oil bioremediation (Fig. 6b). Co has been previously reported to enhance rumen microbial fermentation of substrates in animals (Ryazanov et al. 2022; Wang et al. 2022b; Zhang et al. 2023). Likewise, Co plays a role in the metabolism of methanogenic bacteria (Paulo et al. 2017), which includes species that have been reported to degrade oils in contaminated environments (Sherry et al. 2020; Suda et al. 2021). Fe is another HM which showed a strong positive correlation to methanogenesis. Depending on its form, Fe can promote or hinder methanogenesis in natural anaerobic environments (Baek et al. 2019). Mn and Zn were also found to have a strong and positive correlation to chitinolysis. Chitinolytic bacteria play a significant role in the biodegradation of chitin, one of the most predominant polymers in nature. These microbes synthesize specific enzymes that catalyze the hydrolysis of beta-1,4-glycosidic bonds in low-digestible chitin polymers (Brzezinska et al. 2014; Dhole et al. 2021). The presence of Mn in specific concentrations could enhance the enzyme production of chitinolytic bacteria. Kuddus and Ahmad (2013) observed that the addition of Mn^2+^ in the culture media enhanced chitinase production. However, unlike the results of this study, Zn has been previously reported to inhibit chitinase production (Poria et al. 2021; Ekundayo et al. 2022).

## Limitations of this study

- Sediment samples were collected in only one season. Assessment of changes in microbial diversity and predicted function over multiple seasons would provide new insights into the impact of various environmental factors on the sediment microbial profile.
- Sampling from undisturbed mangrove forests along the Egyptian Red Sea coastline and comparing their sediment microbial profile with the data of this study would lead to the identification of shared and different microbial taxa present in these locations.
- 16S rRNA gene sequencing was used for microbial phylogeny and functional prediction. This approach does not capture the entire DNA of the microbes, allowing for a broader view of the microbes present, their functional genes, and metabolic pathways.
- Whereas Greengenes2 presents a significant advancement in unifying microbial data through a single reference tree, it still presents certain limitations in the taxonomic coverage for less well-characterized environments or organisms not extensively represented in the underlying databases, as well as the inability to classify organisms to a species level.

## Conclusion

This study sheds light on the physiochemical properties, microbial and functional diversity of mangrove sediments along the Egyptian Red Sea coastal areas of Hamata, Mangrove Bay, and Saffaga. Our findings revealed a similarity in the concentration of the measured physiochemical properties among the sediment samples across all depths except for the 30–50 cm depth. Heavy metal analysis showed higher and lower concentrations of copper and iron, lead, manganese, and zinc, respectively, in sediment samples collected in Mangrove Bay compared to those collected in Hamata and Saffaga, thus indicating the role of human activities (i.e., tourism and urbanization) in heavy metal contamination of mangrove ecosystems. All the sediment samples shared similarities in the predominant microbial taxa. Still, they differed in their relative abundance, thus indicating the influence of mangrove species, proximity to the shoreline, and human activity on microbial composition. Furthermore, heavy metals have a significant influence on microbial activity in the mangrove ecosystem; hence, care should be taken in the conservation and restoration of mangrove ecosystems. Further studies are needed to decipher changes in the microbial composition, diversity, and function in different seasons.

## Supplementary information

Below is the link to the electronic supplementary material.

ESM 1 (DOCX 93.0 KB)

ESM 2 (CSV 10.7 KB)

## Data Availability

Microbiological datasets associated with this work have been deposited at the Sequence Read Archive (SRA) of the National Center for Biotechnology Information (NCBI) and are publicly available under the BioProject ID PRJNA1224588. Data sets on the physiochemical properties and heavy metals are publicly available at https://data.mendeley.com/datasets/95zjycf9gh/1.
