# Supplementary material for: Assessment of sediment physiochemical properties, microbial and predicted functional diversity in mangrove eco-restoration sites of Hamata, Mangrove Bay, and Saffaga along the Egyptian Red Sea coast
Source: Environ Sci Pollut Res Int. 2025 Dec 7;32(53):29704–21. doi: 10.1007/s11356-025-37234-1 (PMC12789163; doi:10.1007/s11356-025-37234-1)
Supplement: Supplementary file 1 — (DOCX 93.0 KB) [file 11356_2025_37234_MOESM1_ESM.docx]

**Supplementary Table 1**. Classification and macroelements composition of sediment samples collected along the Egyptian Red Sea Coastal areas

| 0 – 15 cm | | | | |  |  |  |
| --- | --- | --- | --- | --- | --- | --- | --- |
| Sample | **Sand (%)** | **Silt (%)** | **Clay (%)** | **Texture** | **N**  **(mg/kg)** | **P**  **(mg/kg)** | **K (mg/kg)** |
| HA | 4.67^b^ ± 0.76 | 38.50^a^ ± 1.80 | 56.83^a^ ± 1.15 | Clay | 40.33^a^ ± 18.77 | 6.98^b^ ± 0.84 | 191.67^a^ ± 45.17 |
| HR | 3.83^b^ ± 0.58 | 38.67^a^ ± 1.53 | 57.50^a^ ± 1.00 | Clay | 55.33^a^ ± 25.32 | 6.90^b^ ± 1.01 | 82.33^b^ ± 22.19 |
| MA | 72.50^a^ ± 4.09 | 24.50^b^ ± 3.77 | 3.00^c^ ± 0.50 | Loamy Sandy | 79.00^a^ ± 21.66 | 107.00^a^ ± 23.16 | 7.96^c^ ± 0.29 |
| SA | 7.00^b^ ± 1.32 | 42.83^a^ ± 6.43 | 50.17^b^ ± 7.69 | Silty Clay | 32.33^a^ ± 7.57 | 7.11^b^ ± 0.77 | 101.33^b^ ± 23.03 |
| SR | 6.83^b^ ± 0.58 | 43.83^a^ ± 1.89 | 49.33^b^ ± 1.76 | Silty Clay | 46.00^a^ ± 12.12 | 8.32^b^ ± 1.08 | 94.33^b^ ± 7.57 |
| 15 – 30 cm | | | | |  |  |  |
| HA | 3.67^c^ ± 0.76 | 37.50^bc^ ± 0.50 | 58.83^a^ ± 0.58 | Clay | 35.33^b^ ± 2.87 | 7.43^b^ ± 0.55 | 176.00^a^ ± 65.05 |
| HR | 5.00^bc^ ± 0.50 | 40.50^b^ ± 3.28 | 54.50^b^ ± 3.61 | Sandy Loam | 41.67^b^ ± 6.25 | 7.15^b^ ± 0.26 | 112.33^b^ ± 15.70 |
| MA | 67.50^a^ ± 2.00 | 27.83^c^ ± 1.26 | 4.67^d^ ± 0.76 | Silty Clay | 74.67^a^ ± 8.77 | 115.33^a^ ± 29.50 | 8.43^c^ ± 0.55 |
| SA | 7.50^b^ ± 2.00 | 45.00^a^ ± 2.18 | 47.50^c^ ± 2.78 | Silty Clay | 37.67^b^ ± 3.61 | 7.73^b^ ± 0.80 | 69.67^bc^ ± 22.23 |
| SR | 5.50^bc^ ± 0.50 | 42.67^ab^ ± 1.26 | 51.83^b^ ± 1.15 | Silty Clay | 31.67^b^ ± 6.43 | 7.75^b^ ± 0.34 | 81.33^b^ ± 27.54 |
| 30 – 50 cm | | | | |  |  |  |
| HA | 4.17^b^ ± 0.29 | 39.67^a^ ± 2.93 | 56.17^a^ ± 3.21 | Clay | 54.00^a^ ± 4.57 | 8.09^b^ ± 0.63 | 174.33^b^ ± 19.86 |
| HR | 4.50^b^ ± 1.73 | 43.00^a^ ± 1.00 | 52.50^ab^ ± 2.65 | Silty Clay | 45.33^a^ ± 2.27 | 7.45^b^ ± 0.57 | 115.00^b^ ± 5.20 |
| MA | 70.33^a^ ± 6.17 | 26.00^b^ ± 5.27 | 3.67^c^ ± 1.26 | Sandy Loam | 84.00^a^ ± 3.08 | 104.67^a^ ± 9.45 | 10.32^d^ ± 1.93 |
| SA | 7.00^b^ ± 2.18 | 44.00^a^ ± 0.87 | 49.00^b^ ± 1.32 | Silty Clay | 31.33^a^ ± 5.13 | 7.29^b^ ± 0.55 | 356.67^a^ ± 46.33 |
| SR | 4.17^b^ ± 1.53 | 44.50^a^ ± 3.12 | 51.33^b^ ± 1.61 | Silty Clay | 36.00^a^ ± 1.73 | 7.57^b^ ± 0.15 | 78.33^c^ ± 19.76 |
| 50 – 100 cm | | | | |  |  |  |
| HA | 3.17^b^ ± 0.58 | 40.17^b^ ± 0.76 | 56.67^a^ ± 0.29 | Silty Clay | 37.67^b^ ± 1.15 | 7.94^b^ ± 1.09 | 146.33^a^ ± 31.53 |
| HR | 3.50^b^ ± 0.50 | 40.00^b^ ± 2.29 | 56.33^a^ ± 1.76 | Silty Clay | 36.00^b^ ± 7.00 | 7.20^b^ ± 0.40 | 100.67^ab^ ± 21.39 |
| MA | 66.17^a^ ± 2.84 | 29.33^c^ ± 2.75 | 4.50^d^ ± 0.50 | Sandy Loam | 84.00^a^ ± 3.08 | 104.67^a^ ± 9.45 | 10.32^c^ ± 1.93 |
| SA | 5.83^b^ ± 2.31 | 44.83^a^ ± 2.37 | 49.33^c^ ± 1.89 | Silty Clay | 36.67^b^ ± 8.08 | 6.95^b^ ± 0.38 | 82.67^b^ ± 10.69 |
| SR | 5.67^b^ ± 0.29 | 42.00^ab^ ± 1.32 | 52.33^b^ ± 1.15 | Silty Clay | 37.00^b^ ± 2.65 | 7.10^b^ ± 0.24 | 103.67^ab^ ± 39.80 |

Data is presented as mean ± standard deviation (SD). Different upper superscript letters at each depth indicate significant differences at p < 0.05. Sediment samples representing location and species: HA: Hamata, *Avicennia marina*, HR: Hamata, *Rhizophora mucronata*, MA: Mangrove Bay, *Avicennia marina*, SA: Saffaga, *Avicennia marina*, and SR: Saffaga, *Rhizophora mucronata*. N: nitrogen, P: phosphorus, and K: potassium.


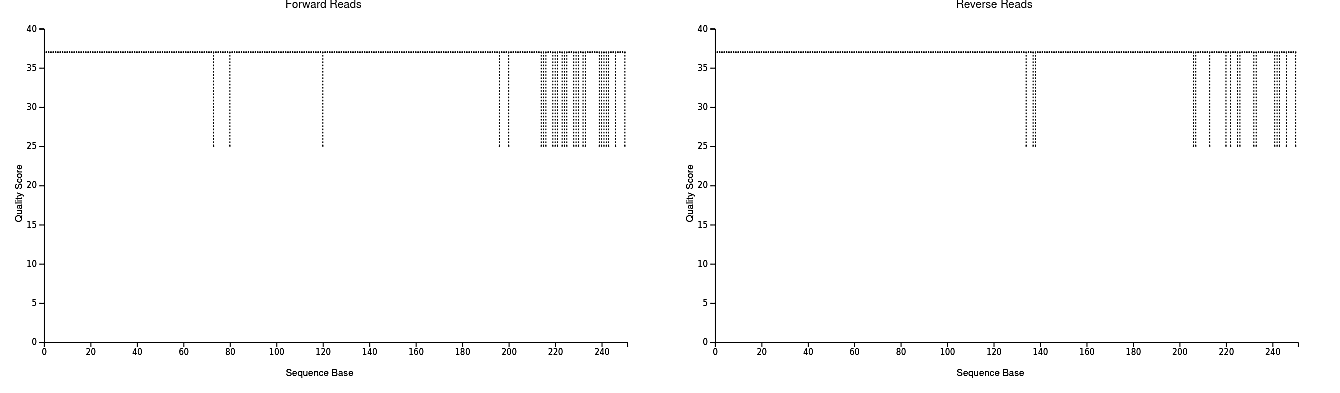


**Supplementary Fig. 1**. Sequence quality of the amplified 16s rDNA samples.
